# Mapping territorial labour-related well-being inequalities in Ecuador: A multivariate geometric approach

**DOI:** 10.1371/journal.pone.0343199

**Published:** 2026-08-31

**Authors:** Bill Jonathan Serrano Orellana

**Affiliations:** Faculty of Business Sciences, Universidad Técnica de Machala, Ecuador; Alexandru Ioan Cuza University: Universitatea Alexandru Ioan Cuza, ROMANIA

## Abstract

This article examines the territorial structure and post-pandemic evolution of labour-related well-being inequalities across Ecuador’s 24 provinces between 2021 and 2024. Using official provincial data from the National Employment, Unemployment and Underemployment Survey (ENEMDU), we constructed an integrated set of eleven indicators capturing employment quantity, job quality, labour income, monetary and multidimensional poverty, and youth labour market exclusion (NEET population aged 15–24). Methodologically, the study adopts a geometric multivariate approach that combines annual HJ-biplots with Partial Triadic Analysis (PTA). This approach allows provinces and indicators to be represented jointly, annual labour-well-being structures to be compared, and a compromise configuration to be extracted that summarises the average post-pandemic profile of the Ecuadorian labour market. The results reveal a stable territorial gradient of labour well-being driven primarily by job quality and income rather than by open unemployment rates. Three persistent provincial clusters emerge: an Amazonian core characterised by cumulative disadvantages in multidimensional poverty, underemployment, and low labour income; an Andean-coastal group with higher levels of formal employment, adequate jobs, and earnings; and an intermediate set of provinces with fragile labour profiles marked by underemployment and youth vulnerability. The PTA confirms a high degree of similarity across annual structures and slow-moving adjustment dynamics, with no evidence of territorial convergence during the period analysed. In addition, the youth NEET indicator defines a distinct axis of labour-related vulnerability, closely associated with unemployment and underemployment but only partially aligned with poverty measures. Overall, the findings highlight the importance of incorporating employment quality and territorial perspectives into assessments of social well-being and underscore the need for labour policies with an explicit territorial and generational focus to address persistent labour-well-being disparities in developing economies.

## Introduction

Over the last decade, labour markets have been exposed to an unusual sequence of global shocks that has reshaped patterns of employment, poverty, and inequality. The 2008 financial crisis left a persistent imprint on young cohorts’ labour-market entry, generating scarring effects on wages and job stability that are still documented across several OECD countries [1,2]. From 2020 onwards, the COVID-19 pandemic triggered a contraction of roughly 3.4% of global GDP and the equivalent loss of hundreds of millions of full-time jobs, alongside a 10% decline in global labour income in the first nine months of the crisis alone [3,4]. The International Labour Organization (ILO) has shown that the impact was not neutral: it was concentrated among groups with higher informality, limited social protection, and occupations not amenable to remote work [3].

In Latin America, where informality and labour precarity were already structural features before the pandemic, these shocks translated into significant setbacks in poverty reduction, inequality, and the availability of quality employment. Joint reports by ECLAC and the ILO document that the region experienced an employment contraction without recent precedent, accompanied by declines in labour-force participation – particularly among women and young people – and an increase in inactivity associated with discouragement [5]. Several studies indicate that the subsequent recovery has been heterogeneous across countries and territories, with job rebounds concentrated in low-productivity and informal segments, thereby limiting the region’s capacity to return to an inclusive growth path [6,7].

This dynamic has unfolded within a pattern of persistent territorial inequalities. The literature on regional disparities in Latin America documents long-standing gaps in productivity, infrastructure access, institutional capacity, and labour opportunities, both across and within countries [8,9]. These inequalities are reflected in the spatial distribution of poverty and in the concentration of low-productivity activities in lagging territories, reproducing the structural heterogeneity characteristic of the region. In particular, recent studies on Brazil, Mexico, and Ecuador confirm that labour precarity and informality tend to cluster in specific subnational spaces, shaping territories of vulnerability where labour and social disadvantages overlap [10,11].

In Ecuador, available evidence points to a cyclical pattern of poverty and inequality, with substantial progress in the decade before 2014 and subsequent reversals linked to falling oil prices, fiscal adjustment, and, more recently, the COVID-19 pandemic [12,13]. Research on financial inclusion and multidimensional poverty suggests that, despite some improvements, territorial and asset-access gaps persist and disproportionately affect rural and Indigenous households, as well as residents of peripheral areas [14,15]. However, much of this work focuses on national aggregate indicators or household microdata without systematically exploiting the provincial dimension of the labour market.

Contemporary debates on job quality emphasize that the unemployment rate is insufficient to describe labour market performance. Through the Decent Work framework, the ILO proposes assessing multiple dimensions simultaneously, including formality, income adequacy, contractual stability, social protection, and working conditions [16,17]. In high-informality contexts such as Ecuador, it is common to observe relatively low unemployment coexisting with a high share of underemployed workers, low-productivity self-employment, or non-affiliation to social security, which complicates the interpretation of traditional aggregate labour market indicators [18].

At the same time, the multidimensional poverty literature has challenged one-dimensional approaches based exclusively on income. Sen’s capability approach and the measurement proposals of Alkire and Foster have consolidated the view that well-being-relevant deprivations span multiple domains – education, health, housing conditions, access to services, and social security – that may or may not coincide with monetary income shortfalls [19,20]. Within this framework, several countries, including Ecuador, have developed official multidimensional poverty indices to guide social policy and monitor progress toward the Sustainable Development Goals [15,21].

The link between job quality and multidimensional poverty is particularly relevant in economies with high informality. Recent studies show that informal employment and underemployment are robustly associated with deprivations in education, health, and housing, as well as with a lower capacity to cope with macroeconomic shocks [22,23]. However, studies that integrate labour indicators and multidimensional poverty within a unified analytical framework remain limited, especially from a territorial and dynamic perspective.

Another critical component of the post-pandemic labour market is the situation of young people who are not in education, employment, or training (NEET). International evidence consistently indicates that prolonged inactivity at the beginning of working life is associated with unstable occupational trajectories, lower future wages, and a higher risk of social exclusion [24,25]. Studies in Europe and the OECD document that NEET youth tend to be concentrated in contexts of low educational quality, segmented labour markets, and weakly inclusive social protection systems [26,27]. In Latin America, NEET rates remain above the average in developed countries and are linked to patterns of informality, teenage pregnancy, and urban violence [28]. Despite this, subnational measurement of NEET rates and their relationship with other employment and poverty dimensions have received limited attention in Ecuador.

From a methodological standpoint, most studies on poverty, inequality, and employment in Ecuador rely on classical econometric techniques (linear or logit regressions and panel models) applied to household survey microdata. These tools are appropriate for identifying individual- or group-level determinants, but they are limited in their ability to capture the joint structure of multiple indicators and their temporal evolution in a subnational setting. In particular, when measures of employment quantity (participation and unemployment), employment quality (adequate employment, formality, and underemployment), youth vulnerability (NEET), and multidimensional poverty (overall and extreme) are analysed simultaneously, complex association patterns emerge that are difficult to interpret using only univariate or bivariate approaches [29].

In this context, multivariate geometric methods provide a powerful framework for jointly representing territories and indicators in low-dimensional spaces. Gabriel’s classical biplot allows the simultaneous visualisation of the rows and columns of a data matrix, facilitating the interpretation of correlations among variables and similarities among observational units [30]. The HJ-biplot, developed by Galindo, generalises this idea by providing optimal representations for both rows and columns, which is particularly useful when territories and well-being dimensions are compared within the same factorial plane [31,32].

However, a purely cross-sectional analysis of a single year does not capture the temporal dimension of labour-market adjustment after the pandemic. Addressing this issue requires methods designed for three-way data structures (territories × indicators × time). Among these, Partial Triadic Analysis (PTA) and the STATIS family of methods are especially suitable because they allow similarity among tables (interstructure), the common or compromise structure of the set (compromise), and the trajectories of each table relative to this consensus to be studied simultaneously [33–35]. Recent applications of PTA in economics and finance – for example, monitoring CAMELS banking indicators under crisis scenarios – illustrate its capacity to extract stable latent factors from multi-temporal matrices and to represent the evolution of economic units graphically within a common space [36].

Despite these methodological advances, to the best of our knowledge no study has combined annual HJ-biplots and PTA to analyse the employment-well-being relationship across Ecuadorian provinces in the post-COVID-19 period. The Ecuador-focused literature has separately addressed multidimensional poverty, financial inclusion, and urban-rural gaps [10,15], while recent analyses of labour precarity and regional inequalities have focused on groups of Latin American countries [6,8]. Yet an integrated characterisation is still missing – one that simultaneously incorporates employment quantity and quality, youth vulnerability, and structural deprivations in well-being within a multivariate and longitudinal framework.

This gap matters for at least three reasons. First, employment and social protection policies are increasingly implemented with a territorial focus; identifying provincial employment-well-being profiles can therefore improve programme targeting and resource allocation [5]. Second, the health crisis and subsequent recovery did not affect all territories uniformly: provinces linked to primary sectors or tourism faced more intense and prolonged shocks, whereas others benefited from a faster rebound in activity. Third, the combination of multidimensional poverty and poor labour conditions generates clusters of persistent disadvantage that may jeopardise progress toward the Sustainable Development Goals related to decent work (SDG 8) and poverty reduction (SDG 1) [37].

Against this backdrop, the central objective of this study is to analyse the structure and territorial evolution of employment-well-being inequalities across Ecuador’s 24 provinces during 2021–2024, using a multivariate geometric approach that combines annual HJ-biplots and Partial Triadic Analysis. Specifically, the study pursues four complementary aims:

1. To characterise, for each year, the joint relationship among indicators of employment quantity (overall labour-force participation rate and unemployment), employment quality (adequate employment, underemployment, formality, and mean labour income), youth vulnerability (NEET aged 15–24), and poverty (overall and extreme income poverty and overall and extreme multidimensional poverty), identifying the main factorial axes that structure the provincial employment-well-being system.
2. To examine the territorial distribution of provinces in annual factorial planes in order to detect spatial patterns of high vulnerability (e.g., in the Amazon region) and higher labour well-being (Andean and coastal provinces with more formal labour markets), as well as changes in these configurations over the post-pandemic period.
3. To synthesise, via PTA, the common structure of the 2021–2024 period by quantifying similarity across the four annual tables, obtaining a compromise matrix that represents the ‘average profile’ of Ecuador’s labour market, and estimating provincial trajectories in the consensus space to identify dynamics of improvement, stagnation, or reversal.
4. To provide methodological evidence on the usefulness of integrating HJ-biplots and PTA for analysing official labour data (ENEMDU), highlighting the advantages of these methods for subnational comparative studies and for monitoring economic recovery processes after major shocks.

Overall, the article’s contribution is primarily applied. Rather than proposing a new method, it develops and illustrates a multivariate geometric framework for territorial monitoring of employment-well-being inequalities using official ENEMDU microdata. The combined use of annual HJ-biplots and Partial Triadic Analysis makes it possible to integrate employment quantity and quality, monetary and multidimensional poverty, and NEET youth vulnerability within a single analytical device, producing comparable maps by province and year. This approach offers a replicable tool for statistical and planning systems to track systematically the evolution of territorial gaps associated with SDGs 1 and 8 in contexts of high structural heterogeneity such as Ecuador. In doing so, the study contributes both to substantive debates on territorial inequalities in employment and well-being in Latin America and to methodological discussions on the use of three-way multivariate techniques in the social sciences. Substantively, it provides a detailed snapshot of how provincial employment-well-being gaps were reorganised in Ecuador during the exit from the health crisis and the transition toward a new economic normal; methodologically, it shows that combining HJ-biplots and PTA helps overcome the limitations of year-by-year analyses by providing a shared reference space in which annual structures and territorial trajectories can be compared.

The remainder of the article is organised as follows.The Literature review presents the conceptual framework on job quality, multidimensional poverty, and territorial inequalities, highlighting its relevance for the Ecuadorian case. The Materials and methods section details the data and methodological strategy, describing the construction of province × indicator × year matrices, the estimation of annual HJ-biplots, and the implementation of Partial Triadic Analysis. The Results section reports the empirical findings of the cross-sectional and longitudinal analyses, the Discussion develops the main interpretations and policy implications, and the Conclusion summarises the principal findings.

### Literature review

#### Employment, multidimensional poverty, and the post-pandemic period in Latin America.

Recent literature agrees that the COVID-19 pandemic deepened pre-existing structural vulnerabilities in Latin American labour markets, particularly in settings characterised by high informality and weak social protection. Recent reports by ECLAC and the ILO show that, after the sharp contraction in 2020, employment recovery has been heterogeneous across countries and territories, with a reallocation toward occupations with lower productivity and weaker labour protection, thereby constraining sustained reductions in poverty and inequality [38,39].

In particular, formal wage employment has expanded less dynamically than informal work and self-employment, and the recovery has been slower for women and young people – groups with a higher prevalence of precarious jobs and lower levels of social protection [40,41].

At the conceptual level, the multidimensional poverty literature has consolidated the view that welfare-relevant deprivations extend beyond monetary income. The capability approach and the Alkire–Foster methodology have been expanded and updated in recent years, with new global and regional measurement exercises incorporating dimensions such as education, health, housing, basic services, and social security [42,43].

For Latin America, recent studies quantify not only the incidence of multidimensional poverty but also vulnerability to falling into poverty following economic shocks, explicitly incorporating links with labour informality and weak social protection. Gallardo et al. [44], using a broad sample of countries in the region, show that the probability of transitioning into multidimensional poverty is significantly higher among households connected to informal occupations, low-productivity self-employment, and youth segments with unstable labour trajectories.

These findings underscore the relevance of approaches that integrate indicators of employment quantity and quality with measures of multidimensional poverty, particularly in economies where open unemployment coexists with large pockets of underemployment, informality, and lack of social protection, as in the case of Ecuador [45,46].

### Job quality, informality, and decent work

The notion of job quality has evolved from predominantly normative approaches toward empirically grounded, multidimensional proposals that combine income, stability, formality, working hours, social security coverage, and working conditions. For Latin America, Sehnbruch et al. [47] propose a multidimensional Quality of Employment (QoE) index for nine countries, showing that job-quality gaps are larger than those observed in employment rates alone and that strong segmentation exists by educational attainment, gender, and region within each country.

In the same vein, Oviedo-Gil et al. [48] examine the relationship between telework and job quality in Argentina, Brazil, and Colombia. They find that the expansion of telework after the pandemic mainly benefited higher-skilled workers and sectors with greater formalization, thereby reinforcing pre-existing gaps [48].

More recently, the IDB’s Better Jobs Index 2024 documents that, even in contexts of GDP and aggregate employment recovery, high levels of informality, underemployment, and insufficient wages persist across much of the region. It also argues that the ‘paradox’ of low unemployment alongside high precarity remains a structural feature [49].

A recurrent conclusion in this literature is that open unemployment loses explanatory power in high-informality settings: unemployment rates may remain moderate while precarious and low-productivity work expands [41,47]. This reinforces the view that labour-market analysis should simultaneously incorporate indicators of adequate employment, underemployment, formality, labour income, and social protection, as proposed in this study for Ecuador’s 24 provinces.

### NEET youth and vulnerable labour trajectories

The situation of young people who are not in education, employment, or training (NEET) has become a key indicator of labour and social vulnerability. A recent review covering more than 150 international studies shows that the risk of being NEET is systematically associated with low educational attainment, precarious socioeconomic conditions, weak institutional quality, and labour-market segmentation [50].

For Latin America, evidence indicates that NEET rates remain above OECD levels and that prolonged inactivity at the start of working life is linked to unstable occupational trajectories, lower wages, and a higher likelihood of informality in adulthood [38,51]. Recent studies combining household surveys with administrative information also show strong territorial differences in the concentration of NEET youth, associated with inequalities in educational quality, access to formal employment opportunities, and exposure to urban violence [41,52].

Although much of this research is conducted at the national or metropolitan level, available evidence suggests that NEET youth tend to cluster in territories facing simultaneous deficits in educational provision, quality employment, and social protection, which is consistent with the notion of ‘territories of vulnerability’ discussed in the regional-inequality literature. Incorporating the NEET indicator within a multivariate framework alongside other labour and poverty dimensions, as this article does, makes it possible to assess whether similar patterns emerge at the provincial level in Ecuador and how they evolved during the 2021–2024 post-pandemic phase.

### Territorial inequalities in employment and well-being

Persistent territorial gaps in productivity, income, and access to services are widely documented across the region. Recent studies on regional inequalities in Latin America emphasise that structural heterogeneity – namely, the coexistence of high-productivity segments with extensive low-productivity sectors – has a spatial expression: natural-resource-intensive activities and informal services tend to concentrate in certain regions, whereas others agglomerate formal employment and higher value-added activities [53,54].

With regard to multidimensional poverty, recent subnational research shows that even when national poverty declines, ‘territorial pockets’ remain where lagging conditions in education, housing, basic services, and quality employment coexist. In Colombia, for example, Sánchez-Torres [55] documents marked intra-regional contrasts in the Pacific region and suggests that national social policies have had limited impact in historically marginalised territories [55].

In Ecuador, the literature highlights the coexistence of provinces with relatively high labour formality and higher incomes, such as Pichincha, Azuay, and Galápagos, with Amazonian and rural territories where underemployment, informality, and multidimensional poverty prevail [45,46]. Recent work on spatial inequalities in the country stresses the need to move toward analyses that combine labour indicators, monetary and multidimensional poverty measures, and access to services, thereby overcoming the sectoral fragmentation of prevailing diagnostics [56].

However, studies that explicitly address the joint structure of these indicators at the provincial level, and their temporal evolution after the pandemic, remain scarce. This study addresses that gap by analysing a province × indicator × year array (2021–2024) integrating participation, unemployment, adequate employment, underemployment, formality, NEET, and monetary and multidimensional poverty.

### Geometric multivariate methods and three-way analysis

From a methodological standpoint, geometric multivariate methods have gained prominence in the social sciences as tools for representing complex sets of indicators in low-dimensional spaces. Gabriel’s classical biplot and, in particular, the HJ-biplot developed by Galindo allow the simultaneous visualisation of observational units and variables, optimising the representation of both within the same factorial plane. A recent review published in Mathematics synthesises the theoretical foundations of the HJ-biplot and documents its rapid expansion in studies of education, health, finance, and labour markets [30,31,57].

Recent empirical applications highlight the usefulness of the HJ-biplot for analysing complex socioeconomic systems, including educational inequalities, financial performance, and sectoral employment patterns [58].

When data exhibit a three-way structure (units × variables × time periods or scenarios), the STATIS family – and within it, Partial Triadic Analysis (PTA) – provides particularly suitable tools. Abdi et al. [59] show that these methods make it possible to decompose total variation into three components: similarity among tables (interstructure), the common structure or compromise matrix, and table-specific features (intrastructure), thereby offering a unified framework for comparative analyses across multiple temporal or spatial cuts.

Recent Q1-journal applications illustrate the potential of PTA and related three-way techniques in contexts of sustainable development and inequality. Medina-Hernández et al. [60] use a STATICO-PTA approach to study co-variation between gender indicators and other Sustainable Development Goals, showing how the compromise matrix summarises robust patterns of association among gender equity, poverty, and health. In the environmental domain, articles published in Water and Scientific Reports apply PTA and other three-way methods to analyse the joint dynamics of water quality, anthropogenic pressure, and climate, underscoring the capacity of these techniques to extract stable latent factors from multi-table time series [61].

In sum, recent literature suggests that combining HJ-biplots and PTA constitutes a robust methodological strategy for studying complex socioeconomic systems in which the aim is to characterise both the multivariate structure of each period and the joint evolution over time. This study builds on that tradition by applying annual HJ-biplots to employment and well-being indicators for Ecuadorian provinces and by using PTA to extract a 2021–2024 consensus structure and identify provincial trajectories in the employment-well-being space.

## Materials and methods

The methodology is organised into three complementary stages:

(i) construction and standardisation of the province × indicator × year matrices;
(ii) cross-sectional analysis using annual HJ-biplots to characterise the employment-well-being structure in each time slice; and
(iii)   longitudinal analysis using Partial Triadic Analysis (PTA), which synthesises the common structure of the 2021–2024 period and identifies provincial trajectories.

This combination enables an integrated reading of the phenomenon, both static (year by year) and dynamic (across years).

This study examines the employment-well-being relationship at the subnational level in Ecuador using a multivariate approach applied to the 24 provinces over 2021–2024, a period covering the exit from the health crisis and the transition toward economic recovery. Official data come from INEC’s National Survey of Employment, Unemployment and Underemployment (ENEMDU) and its associated tabulations and methodological documents (accessed on 21 November 2025).

The analysis begins in 2021 rather than 2020 because 2020 reflected the acute pandemic shock and exceptionally disrupted labour-market conditions, which may reduce interannual comparability. Focusing on 2021–2024 allows the study to examine the post-pandemic recovery phase under more stable conditions. The combined use of HJ-biplot and PTA is justified by the study’s objective of analysing the joint structure of eleven interrelated indicators across 24 provinces and four annual slices. Unlike conventional panel or spatial econometric approaches, which are better suited to testing specific causal hypotheses, this multivariate geometric strategy provides a simultaneous representation of provinces and indicators and a common factorial space for comparing annual territorial trajectories.

We constructed a province × indicator × year array with eleven comparable variables: overall labour-force participation rate, unemployment, adequate employment, underemployment, formality, NEET (ages 15–24), income poverty (overall and extreme), multidimensional poverty (overall and extreme), and mean labour income. The choice of the 2021–2024 period provides a clear basis for dynamic assessment.

We did not alter the original orientation of the indicators. Although the literature often classifies some indicators as positive or negative, their empirical behaviour in Ecuador does not follow a single well-being-precarity gradient. In a context of structural informality and low open unemployment, imposing polarity inversions would distort the factorial geometry of the HJ-biplots. Therefore, all variables were retained exactly as reported in official statistics, and only column-wise standardisation was applied.

To ensure methodological consistency, we adopted INEC’s operational definitions for adequate employment, underemployment, and formality/informality, as well as the official methodologies for income poverty and multidimensional poverty. The NEET (15–24) indicator was estimated in accordance with international standards.

The analysis was conducted at two levels. First, annual HJ-biplots characterise the multivariate structure of each year by identifying relationships among indicators and provincial contrasts. Second, PTA integrates the four annual slices into a common synthesis: it assesses similarity among years (interstructure), derives the period’s consensus geometry (compromise), and projects each year onto that common space (trajectories). In this way, the two methods are complementary: the HJ-biplot provides a cross-sectional view, whereas PTA provides a longitudinal view of the phenomenon.

Using these series, we estimated annual HJ-biplots that jointly represent provinces and indicators for each year and performed a Partial Triadic Analysis (PTA) to extract the common structure of the period, weight annual matrices through compromise weights, and identify provincial trajectories (improvement, stagnation, or deterioration) within the shared factorial space. PTA quantifies the inertia shared across the four years, as well as year-specific features, offering a more stable dynamic reading than isolated year-by-year comparisons.

### Variables and operationalization

#### Analytical approach and domain selection.

The analysis focuses on provincial indicators capturing employment quantity and quality, youth vulnerability, and structural deprivations in well-being. Variable selection followed three criteria: (i) conceptual relevance within the decent work and multidimensional poverty frameworks, (ii) annual availability and comparability over 2021–2024, and (iii) the capacity to generate robust latent axes in the annual HJ-biplots and to describe provincial trajectories through PTA.

For each year t, we constructed a province × indicator matrix for the 24 provinces and 11 variables included in the analysis, denoted X^(t) ∈ R^(24 × 11). Table 1 reports the abbreviation, full name, and operational definition of each variable used in the analysis.

**Table 1:** Abbreviation, full name, and operational definition of the indicators included in the province × indicator × year array.

Variable	Full name	Definition
TPG	Overall labour force participation rate	Percentage of the working-age population that is employed or actively seeking employment.
DES	Unemployment rate	Percentage of the labour force that is without work, available for work, and actively seeking employment.
EA	Adequate employment rate	Employed individuals with earnings ≥ the minimum wage and working ≥ 40 hours per week; or working < 40 hours per week with no desire and no availability to work additional hours.
SUB	Underemployment rate	Employed individuals with earnings below the minimum wage and/or working fewer hours than desired, who are available to work additional hours.
FOR	Formal employment share	Percentage of employed workers in establishments registered with a tax identification number (RUC), used as a proxy for formality.
NEET_15_24	NEET rate (ages 15–24)	Percentage of young people aged 15–24 who are not in education, not in training, and not in employment.
POB_ing	Income poverty rate	Percentage of the population living below the official income poverty line.
POB_ext	Extreme income poverty rate	Percentage of the population living below the official extreme income poverty line.
PM	Multidimensional poverty rate	Percentage of the population experiencing simultaneous deprivations according to the Alkire–Foster/INEC methodology.
PM_ext	Extreme multidimensional poverty rate	Percentage of the population with a deprivation intensity above the extreme threshold.
ING	Average labour income	Average monthly labour income of employed individuals, in current USD.
		

### HJ-biplot analysis

Annual HJ-biplots were estimated for 2021, 2022, 2023, and 2024 to describe the cross-sectional structure of the employment-well-being system in each year. This method was selected because it provides a balanced joint representation of provinces and indicators in the same factorial plane, facilitating the identification of territorial similarities, the association structure among variables, and the main dimensions underlying labour-related well-being. Following Gabriel’s biplot framework [30] and the symmetric HJ scaling proposed by Galindo-Villardón [31], provinces are represented as points and indicators as vectors in a reduced Euclidean space. In this representation, distances between provinces approximate similarity in multivariate profiles, whereas angles between vectors summarise the association structure among indicators.

### Data structure and preprocessing

Official provincial data from ENEMDU/INEC were organised, for each year, into a 24 × 11 province × indicator matrix, with provinces in rows and the eleven labour-market and well-being indicators in columns. The same structure was maintained across the four annual tables to ensure direct comparability between years. All variables were standardised by columns before the HJ-biplot analysis.

### Variable preprocessing

All indicators were standardised by columns within each year (z-scores). This step neutralises differences in measurement scales, preserves the original orientation of the official indicators, and prevents variables expressed in larger numerical ranges from dominating the factorial solution.

### Definition of temporal slices

One HJ-biplot was estimated for each year, allowing provincial configurations to be compared across four sequential phases.

Initial recovery (2021). This was the first year after the lifting of the main public-health restrictions and provides the baseline configuration of the multivariate space.

Reactivation (2022). This second year reflects a context of progressive reactivation and allows the interannual coherence and stability of the structure identified in 2021 to be assessed.

Adjustment (2023). This stage captures the persistence or reorientation of the main latent axes and possible provincial shifts relative to the previous year.

Consolidation (2024). This most recent slice is used to examine convergence, divergence, or consolidation of factorial patterns at the end of the study period.

**Fig 1:**
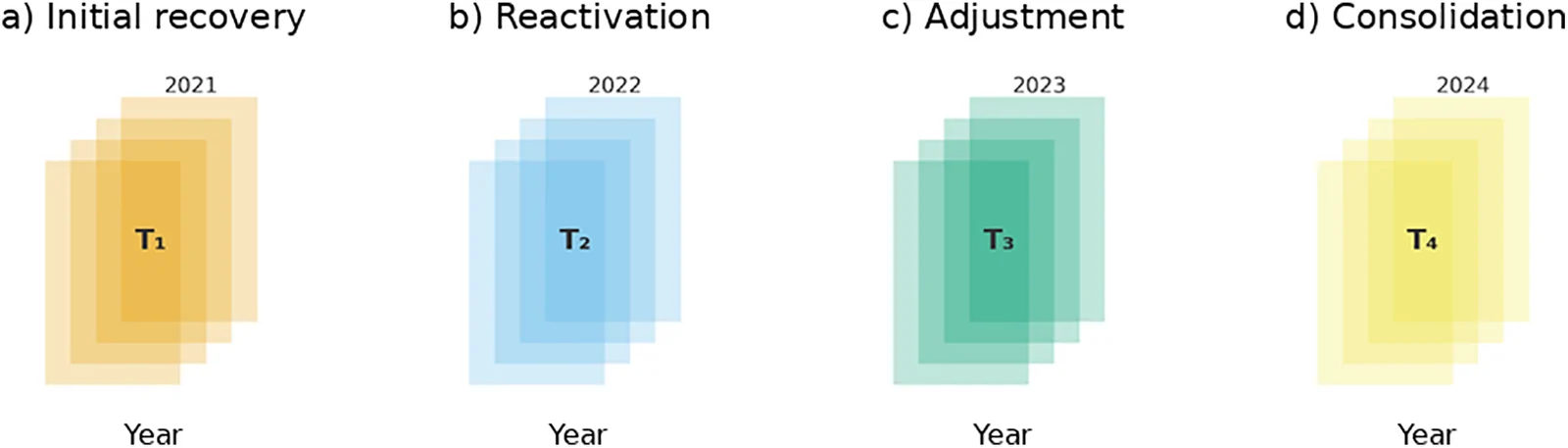
Analytical periods included in the study (2021–2024).

The four temporal slices considered in the analysis are summarized in Fig 1.

For each year t ∈ {2021, 2022, 2023, 2024}, the standardised province × indicator matrix was analysed using the HJ-biplot. Singular value decomposition was used to obtain the factorial coordinates of provinces and indicators under symmetric scaling. The interpretation focused on explained inertia, quality of representation (cos^2^), contributions (CTR), and the substantive meaning of the main axes.

### Estimation and interpretation criteria

The annual HJ-biplots were estimated in MultBiplot software and cross-checked in R. In practical terms, the method relies on the singular value decomposition of each standardised matrix to obtain joint coordinates for provinces and indicators under symmetric scaling.

Interpretation focused on the first two axes whenever they provided a stable summary of total inertia. Axis labelling was based on the signs and magnitudes of the variable vectors, while quality-of-representation (cos^2^ and contribution (CTR) measures were used to identify the provinces and indicators that most strongly defined each dimension.

This level of detail is sufficient for replication and is more appropriate for a broad interdisciplinary readership than a full textbook derivation of the HJ-biplot formulae.


$$\begin{array}{c} X=\left[\;x_{ij}\;\right]\in R^{n\times p} \end{array}$$

where X is the column-standardised data matrix, with


$$\begin{array}{c} x_{ij}=\frac{X_{ij}^{\text{orig}}-\overset{―}{X_{\cdot j}}}{s_{j}},\;\overset{―}{X_{\cdot j}}=\frac{\text{1}}{n}\sum_{i=\text{1}}^{n}X_{ij}^{\text{orig}},\;s_{j}=\sqrt{\frac{\text{1}}{n-\text{1}}\sum_{i=\text{1}}^{n}{\left(X_{ij}^{\text{orig}}-\overset{―}{X_{\cdot j}}\right)}^{\text{2}}} \end{array}$$

Under this standardisation, each column has mean 0 and variance 1. The use of z-scores prevents heterogeneous measurement scales from dominating the solution.

The analysis was conducted in the standard Euclidean space, using symmetric inner products on the standardised matrix X. Accordingly, distances between provinces correspond to Euclidean distances in the principal-component space, while angles between variable vectors approximate linear correlations among indicators. This choice of metric is consistent with the classical HJ-biplot formulation and preserves the geometric properties derived from the singular value decomposition.


$$\begin{array}{c} X=U\;D\;V^{⊤},\;U\in R^{n\times r},\;V\in R^{p\times r},\;D=\text{diag}\left(\sigma_{\text{1}},\ldots ,\sigma_{r}\right),\;\sigma_{\text{1}}\geq \cdots \geq \sigma_{r}>\text{0} \end{array}$$

$$\begin{array}{c} =\text{rank}(X),\;U^{T}U=I_{r},\;V^{T}V=I_{r} \end{array}$$

$$\begin{array}{c} X{|}_{F}^{\text{2}}=\sum_{k=\text{1}}^{r}\sigma_{k}^{\text{2}} \end{array}$$

$$\begin{array}{c} R=UD^{\alpha },\;C=VD^{\;\text{1}-\alpha } \end{array}$$

Then,


$$\begin{array}{c} X\approx R\;C^{⊤},\;x_{ij}\approx r_{i}^{⊤}\;c_{j}, \end{array}$$

where $r_{i}^{T}$is the i-th row of R(coordinates of province i) and $c_{j}$ is the j-th row of C (coordinates of variable j).

Let $R_{m}$ and C_m_ denote the matrices obtained by retaining the first m columns of R and C. The m-dimensional bilinear approximation is


$$\begin{array}{c} X\approx R_{m}\;C_{m}^{⊤}=\sum_{k=\text{1}}^{m}\sigma_{k}\;u_{k}\;v_{k}^{⊤}, \end{array}$$

which is analogous to PCA reconstruction. The proportion of variance explained by the (1–2) plane is:


$$\begin{array}{c} \text{Va}{\text{r}}_{\left(\text{1},\text{2}\right)}=\frac{\sigma_{\text{1}}^{\text{2}}+\sigma_{\text{2}}^{\text{2}}}{\sum_{k=\text{1}}^{r}\sigma_{k}^{\text{2}}} \end{array}$$

**• Correlation between variables**: the angle between vectors $c_{j}$ and $c_{j^{\prime }}$ approximates the sign and magnitude of corr $\left(X_{\cdot j},\;X_{\cdot j^{\prime }}\right)$ (small angles indicate strong positive correlation; angles close to 180° indicate negative correlation; angles near 90° indicate weak correlation).


$$\begin{array}{c} \text{With}R_{m}=\left[\;r_{ik}\;\right]\text{and }C_{m}=\left[\;c_{jk}\;\right] \end{array}$$

$$\begin{array}{c} {\text{cos}}^{\text{2}}(i)=\frac{\sum_{k=\text{1}}^{m}r_{ik}^{\text{2}}}{\sum_{k=\text{1}}^{r}r_{ik}^{\text{2}}},\;{\text{cos}}^{\text{2}}(j)=\frac{\sum_{k=\text{1}}^{m}c_{jk}^{\text{2}}}{\sum_{k=\text{1}}^{r}c_{jk}^{\text{2}}} \end{array}$$

$$\begin{array}{c} \text{CT}{\text{R}}_{\text{i}\to \text{k}}=\frac{r_{ik}^{\text{2}}}{\sum_{i^{\prime }=\text{1}}^{n}r_{i^{\prime }k}^{\text{2}}},\;\text{CT}{\text{R}}_{\text{j}\to \text{k}}^{\left(\text{var}\right)}=\frac{c_{jk}^{\text{2}}}{\sum_{j^{\prime }=\text{1}}^{p}c_{j^{\prime }k}^{\text{2}}} \end{array}$$

### Selection of dimensionality

The number of retained dimensions m is chosen based on: (i) a scree plot of $\sigma_{K}^{\text{2}}$, (ii) a cumulative inertia threshold, and (iii) interpretive stability—i.e., whether cos² and CTR patterns yield a stable and coherent reading of the axes.

1. Singular value decomposition:$X^{\left(t\right)}=U\;D\;V^{⊤}$
2. Dimension selection: m = 2 or m = 3 based on the elbow criterion, explained inertia, and interpretive stability.
3. Visualisation: simultaneous projection of provinces and variables on the (1–2) plane.

### Partial triadic analysis (PTA): formulation, construction, and interpretation

PTA was used because the data form a three-way array with identical rows (provinces) and columns (indicators) across years. The method provides a longitudinal complement to the annual HJ-biplots by identifying the common structure of the 2021–2024 period and projecting each annual table onto the same consensus space.

Following the PTA framework developed for multi-table analysis, the procedure was implemented in three stages: interstructure, compromise, and intrastructure. Interstructure quantifies the similarity among annual tables, the compromise synthesises the average post-pandemic structure, and the intrastructure projects each year onto the consensus space to recover provincial trajectories.

PTA makes it possible to identify dynamic trends and common structures across years, providing a fine-grained account of how provincial employment-well-being profiles evolved in Ecuador during the exit from the pandemic and the subsequent recovery phase.

From this point onward, the province × indicator × year array is analysed formally using PTA, following its three canonical phases.

Partial Triadic Analysis (PTA), introduced within the French school of data analysis by Thioulouse and Chessel [62], is a method for analysing three-way data structures viewed as a sequence of tables sharing the same rows and columns (e.g., provinces × indicators observed over multiple years). PTA belongs to the family of k-table methods and proceeds through three canonical stages: interstructure, compromise, and intrastructure/trajectories.

PTA therefore makes it possible to identify dynamic trends and common structures across years, providing a fine-grained account of how provincial employment-well-being profiles evolved in Ecuador during the exit from the pandemic and the subsequent recovery phase.

### Data structure

Using official ENEMDU/INEC provincial data, the four annual matrices were arranged into a 24 × 11 × 4 array with identical rows and columns across years. Each annual block is denoted X^(t) ∈ R^(I × J). Within each year, variables were standardised by columns so that interannual comparisons would reflect structure rather than scale differences.

### Definition of periods

PTA was implemented using the four annual slices shown in Fig 1: 2021 (initial recovery), 2022 (reactivation), 2023 (adjustment), and 2024 (consolidation). This annual partition allows the compromise analysis to summarise the shared post-pandemic structure while preserving moderate year-to-year variation.

### Notation and preprocessing

Reporting focused on RV coefficients, compromise weights, explained inertia, and the interpretation of provinces and indicators in the consensus space. As in the HJ-biplot stage, the reading of the axes was supported by contributions and squared cosines, whereas provincial trajectories were assessed through the direction and magnitude of movement across years.

This condensed presentation preserves reproducibility while keeping the methods section focused on why PTA is appropriate for a province × indicator × year array and how it complements the annual HJ-biplots.

### Interstructure, compromise, and trajectories

PTA was implemented in its three canonical stages. First, interstructure was used to quantify similarity among annual tables through RV coefficients and associated weights, thereby identifying the years that best represent the shared post-pandemic pattern. Second, the compromise matrix was computed as the weighted synthesis of the four annual tables and analysed in a reduced factorial space. Third, each annual table was projected onto that consensus space to reconstruct provincial trajectories over 2021–2024.


$$\begin{array}{c} g_{ts}={\left\langle X^{\left(t\right)},X^{\left(s\right)}\right\rangle }_{F}=\;\text{tr}\left(X^{\left(t\right)}X^{(s)⊤}\right),\;\text{RV}\left(X^{\left(t\right)},\;X^{\left(s\right)}\right)=\frac{\text{tr}\left(X^{\left(t\right)}X^{(t)T}X^{\left(s\right)}X^{(s)T}\right)}{\left|\right|X^{\left(t\right)}X^{(t)T}{\left|\right|}_{F}\;\left|\right|X^{\left(s\right)}X^{(s)T}{\left|\right|}_{F}} \end{array}$$

$$\begin{array}{c} X^{\left(⋆\right)}=\sum_{t=\text{1}}^{T}\alpha_{t}\;X^{\left(t\right)}. \end{array}$$

$$\begin{array}{c} X^{\left(⋆\right)}=U^{\left(⋆\right)}\;D^{\left(⋆\right)}\;{\left(V^{\left(⋆\right)}\right)}^{⊤}, \end{array}$$

The inertia explained by the (1–2) plane is:


$$\begin{array}{c} \frac{\sigma_{\text{1}}^{(⋆)\text{2}}+\sigma_{\text{2}}^{(⋆)\text{2}}}{\sum_{k}\sigma_{k}^{(⋆)\text{2}}} \end{array}$$

The compromise matrix provides the consensus configuration of the employment-well-being system, whereas the annual projections make it possible to trace moderate improvements, stagnation, or reconfiguration processes at the provincial level.

### Methodological synthesis

Overall, the methodology combines cross-sectional multivariate analysis (HJ-biplot) and longitudinal multi-table analysis (PTA) to study the employment-well-being relationship in Ecuador. Annual HJ-biplots reveal year-specific structural patterns, whereas PTA identifies the consensus structure and provincial trajectories over time. This approach is descriptive and exploratory rather than causal, and it is intended to complement, rather than replace, conventional econometric analysis.

### Computational considerations and reproducibility

All analyses were implemented in R (version 4.4.3). Data preprocessing and construction of the province × indicator × year array were carried out using functions from the tidyverse ecosystem. Annual HJ-biplots were generated using MultBiplot software, developed by J. L. Vicente-Villardón, and cross-validated with custom routines in R. Partial Triadic Analysis (PTA) was conducted using the pta() and kplot() functions from the ade4 package. The standardised provincial dataset and variable dictionary are provided as Supporting Information, ensuring transparency and facilitating reproducibility of the analytical workflow.

## Results

This section reports the empirical findings at two complementary levels: (i) a cross-sectional annual characterisation using HJ-biplots and (ii) a longitudinal synthesis using Partial Triadic Analysis (PTA) for 2021–2024. Interpretation and policy implications are developed in the Discussion section.

### Cross-sectional analysis: annual HJ-biplots (2021–2024)

#### Overall axis structure.

Across the four years analysed (see Table 2), the first two axes explain between 69% and 79% of total variability, ensuring a robust representation of the provincial employment-well-being system. In all cases, Axis 1 consistently reproduces the formality-income gradient in contrast to poverty and structural deprivations, whereas Axis 2 captures a complementary dimension associated with underemployment, youth disadvantage (NEET), and labour-force participation.

**Table 2:** Eigenvalues and explained variance (HJ-biplots, 2021–2024).

Year	2021	2022	2023	2024	2021	2022	2023	2024
Axis	Eigenval.	Eigenval.	Eigenval.	Eigenval.	Expl. Var.	Expl. Var.	Expl. Var.	Expl. Var.
Axis 1	133.964	155.816	154.062	155.042	52.95	61.587	60.894	61.281
Axis 2	40.968	48.725	42.093	43.791	16.19	19.26	16.64	17.31
Axis 3	25.566	27.557	23.57	27.132	10.11	10.89	9.32	10.72
Axis 4	21.762	8.605	11.538	14.383	8.60	3.40	4.56	5.69
Axis 5	14.973	6.922	9.966	6.007	5.92	2.74	3.94	2.37
Axis 6	7.535	2.687	6.785	2.876	2.98	1.06	2.68	1.14
Axis 7	4.719	1.216	2.43	1.88	1.87	0.48	0.96	0.74
Axis 8	2.062	0.752	1.738	1.016	0.82	0.30	0.69	0.40
Axis 9	0.871	0.363	0.341	0.596	0.34	0.14	0.14	0.24
Axis 10	0.489	0.187	0.296	0.166	0.19	0.07	0.12	0.07
Axis 11	0.091	0.172	0.181	0.112	0.04	0.07	0.07	0.04

Despite minor year-to-year variations in magnitudes and loadings, the overall orientation of the vectors remains internally coherent, indicating stability in the factorial structure of Ecuador’s labour market over 2021–2024.

### Evolution of indicators along the axes

Across all years analysed, adequate employment (EA), formality (FOR), and labour income (ING) consistently project onto the positive pole of Axis 1. Their contributions – exceeding 800 × 10 ⁻ ^3^ in most periods – confirm that this axis stably summarises a labour well-being pattern associated with formal labour-market attachment and higher income levels. In contrast, multidimensional poverty (PM), extreme multidimensional poverty (PM_ext), and income poverty (POB_ing, POB_ext) remain systematically located on the negative pole of the same axis, with contributions above 850 × 10 ⁻ ^3^. This stability indicates that Axis 1 represents a robust structure opposing high socioeconomic vulnerability to contexts characterised by greater formality and labour security.

Axis 2 exhibits greater interannual variation; nevertheless, its defining patterns remain clearly identifiable. Underemployment (SUB) and the share of young people outside the education-employment system (NEET) tend to be positioned on the positive pole, reflecting dynamics of precarity, instability, and youth disadvantage. On the negative pole, the overall labour-force participation rate (TPG) and labour income (ING) consistently project together, capturing a component associated with labour-market dynamism and stronger workforce integration.

Taken together, and as shown by the variable contributions reported in Table 3, Axis 1 functions as a structural gradient between poverty and formality, whereas Axis 2 captures labour-market heterogeneity and the levels of youth vulnerability that shape territorial differences over the period under study.

**Table 3:** Variable contributions.

Year	2021		2022		2023		2024	
Variables	Axis 1	Axis 2	Axis 1	Axis 2	Axis 1	Axis 2	Axis 1	Axis 2
TPG	335	554	512	381	538	333	457	425
DES	509	13	349	27	348	31	189	100
EA	655	12	899	34	879	29	923	12
SUB	0	72	2	628	23	666	0	744
FOR	837	95	843	102	869	63	839	81
NEET_15_24	342	574	280	515	481	291	508	134
POB_ing	770	84	855	21	820	16	818	43
POB_ext	133	101	768	3	751	8	697	2
PM	897	9	943	1	938	1	933	0
PM_ext	841	5	880	14	655	7	914	22
ING	505	264	443	392	397	386	463	344

### Comparative provincial distribution

Consistently across the four years analysed, three clearly differentiated spatial configurations emerge, revealing stable structural patterns in the territorial distribution of well-being and labour vulnerability.

First, the Amazonian block – comprising Napo, Orellana, Pastaza, and Morona Santiago – is systematically located at the negative extreme of Axis 1. Its position is closely aligned with the vectors for multidimensional poverty, extreme poverty, and structural deprivations, reflecting a persistent accumulation of socioeconomic disadvantages. In addition, these provinces exhibit the highest annual contributions on this axis, confirming their central role in shaping the territorial gradient. Their recurrent location indicates that, beyond cyclical fluctuations, the Ecuadorian Amazon continues to constitute the structural core of socioeconomic vulnerability in Ecuador, maintaining a marked distance from other territories.

Second, an Andean-coastal block with higher levels of labour well-being – comprising Pichincha, Galápagos, Guayas, Azuay, and El Oro – appears consistently on the positive pole of Axis 1 throughout the period. These provinces are associated with higher levels of formality, adequate employment, and labour income, forming a sustained territorial pattern of favourable labour-market insertion. Within this group, Galápagos and Pichincha stand out because they display the highest coordinates in all years after 2021, consolidating their position as the territories with the strongest relative labour and economic indicators. This behaviour highlights the persistence of regional inequalities and the consolidation of structural well-being poles.

Finally, a set of provinces with intermediate patterns – or patterns highly dependent on the dynamics captured by Axis 2 – is identified, notably Santa Elena, Manabí, Esmeraldas, and Los Ríos. Although they do not occupy extreme positions on Axis 1, in all years they tend to project toward the positive pole of Axis 2, where vectors associated with underemployment and the share of NEET youth are concentrated. While their positions exhibit greater interannual variability than those of the previous blocks, these provinces play a key role in explaining the second component: they represent territories where labour-market insertion is more unstable and where persistent dynamics of youth disadvantage and precarity are observed. This pattern suggests that Axis 2 captures not only labour-market heterogeneity but also processes of incomplete transition toward more integrated labour markets.

Taken together, and considering the provincial contributions reported in Table 4, these three territorial blocks confirm the existence of a robust spatial structure that remains stable over time, clearly differentiating territories facing persistent vulnerability, provinces with consolidated labour well-being, and intermediate zones shaped by specific dynamics of precarity and youth disadvantage.

**Table 4:** Provincial contributions.

Year	2021		2022		2023		2024	
Provinces	Axis 1	Axis 2	Axis 1	Axis 2	Axis 1	Axis 2	Axis 1	Axis 2
**Azuay**	686	96	625	206	599	171	744	114
**Bolivar**	580	26	121	58	46	8	222	38
**Cañar**	349	114	305	1	104	267	305	254
**Carchi**	8	207	394	372	10	28	14	208
**Chimborazo**	739	146	635	11	440	176	420	236
**Cotopaxi**	110	511	194	359	579	77	602	229
**El Oro**	893	14	731	117	755	99	875	15
**Esmeraldas**	6	461	3	440	6	388	10	415
**Galapagos**	649	221	551	395	604	314	616	268
**Guayas**	616	121	789	99	201	40	688	193
**Imbabura**	581	274	446	97	538	110	208	210
**Loja**	1	31	134	6	239	124	448	1
**Los Rios**	28	363	116	394	409	359	607	157
**Manabi**	24	399	152	661	259	503	241	553
**Morona Santiago**	948	0	955	1	930	0	844	43
**Napo**	954	11	890	2	951	1	943	4
**Orellana**	871	37	875	102	959	8	940	7
**Pastaza**	848	83	864	105	880	97	815	97
**Pichincha**	882	7	829	20	793	56	755	44
**Santa Elena**	167	572	179	666	171	726	78	763
**Santo Domingo**	641	37	535	205	456	160	336	333
**Sucumbios**	286	351	284	2	424	32	354	69
**Tungurahua**	19	299	27	353	73	205	148	167
**Zamora Chinchipe**	0	126	417	234	234	127	283	225

### Longitudinal integration of the 2021–2024 biplots

**Fig 2:**
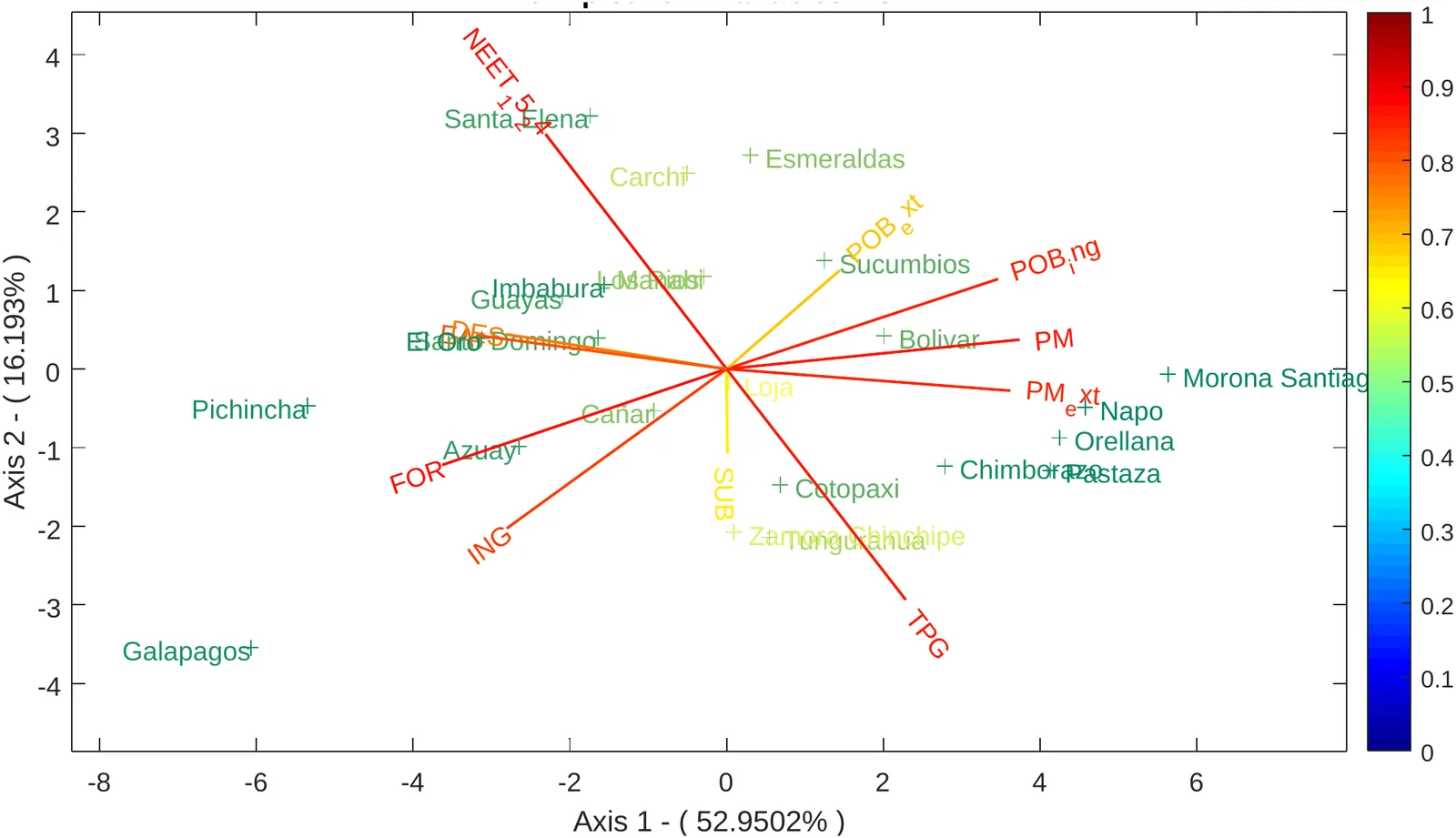
HJ-biplot of provinces and labour-market indicators (Ecuador, 2021). The factorial plane (1–2) explains 69.14% of total inertia.

**Fig 3:**
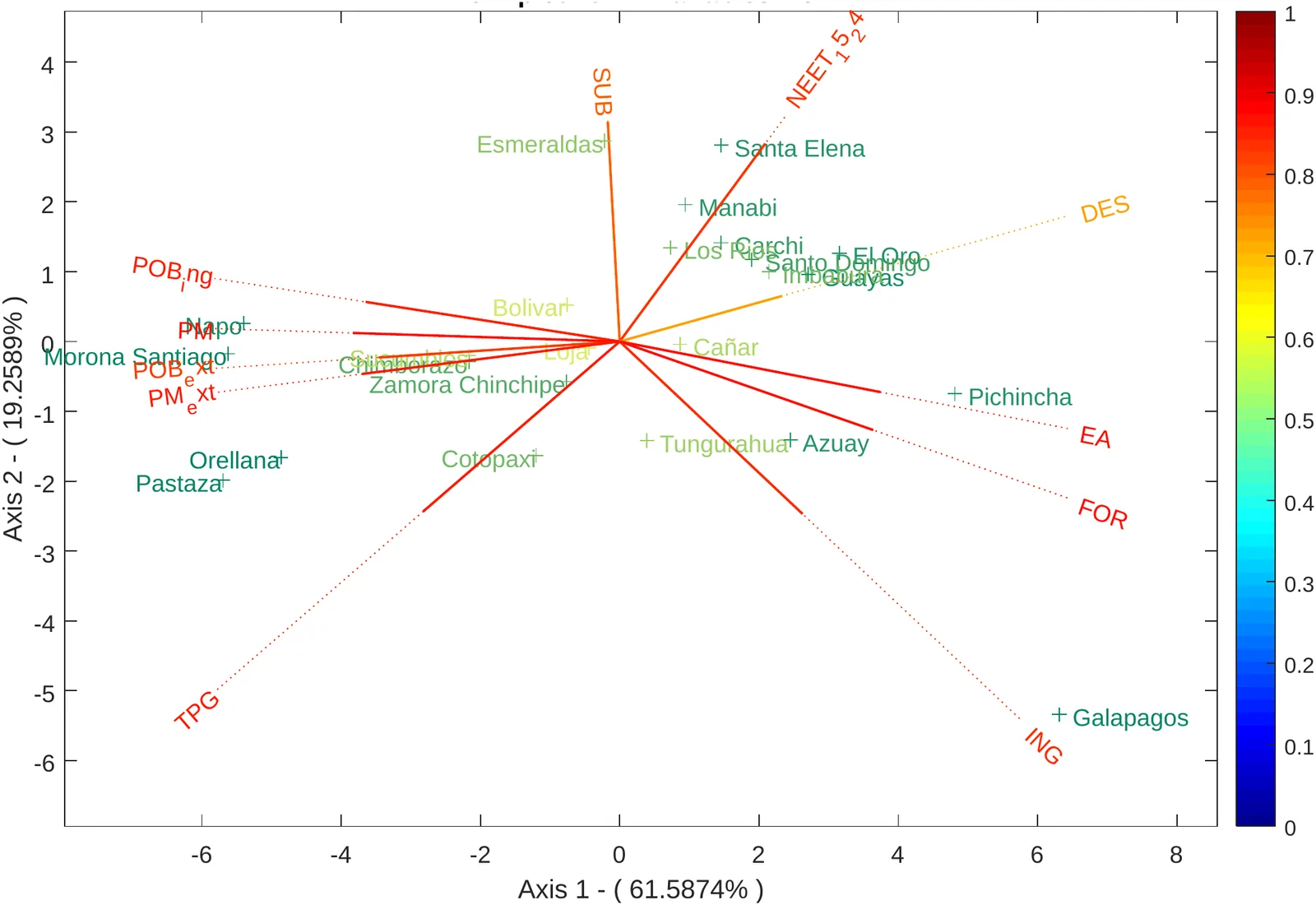
HJ-biplot of provinces and labour-market indicators (Ecuador, 2022). Axes 1–2 explain 80.85% of total inertia.

**Fig 4:**
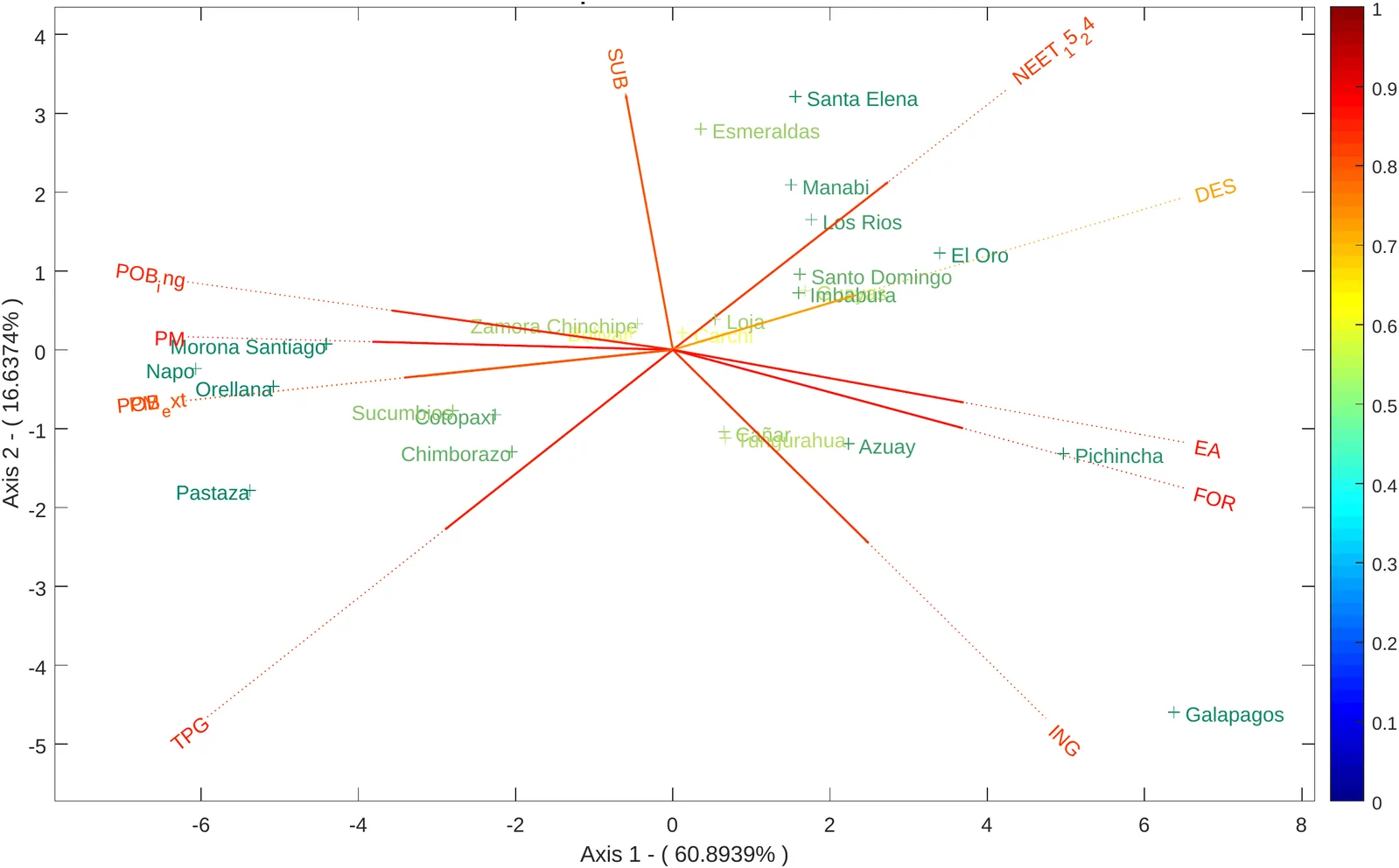
HJ-biplot of provinces and labour-market indicators (Ecuador, 2023). Axes 1–2 explain 77.53% of total inertia.

**Fig 5:**
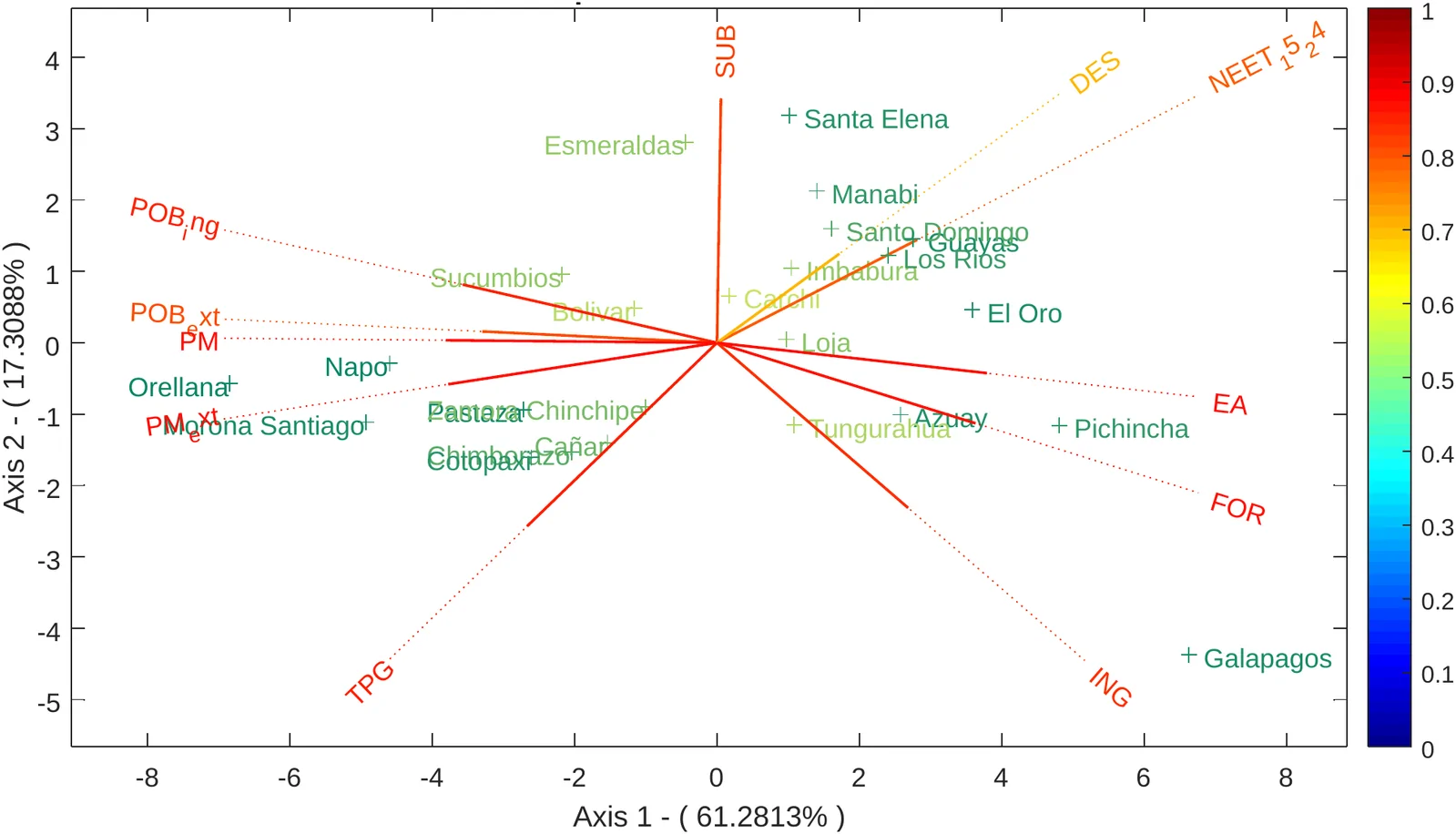
HJ-biplot of provinces and labour-market indicators (Ecuador, 2024). Axes 1–2 explain 78.59% of total inertia.

The comparative analysis of the four HJ-biplots (see Fig 2, Fig 3, Fig 4, Fig 5) for 2021, 2022, 2023, and 2024 reveals a highly stable spatial structure over time, in which the axes retain their interpretive meaning and provinces preserve consistent positioning patterns. Despite minor variations in the magnitude of some coordinates, the overall configuration of the factorial space remains virtually unchanged.

Across the four years, Axis 1 maintains its role as a gradient contrasting poverty-vulnerability with formality-labour well-being. Variables associated with adequate employment (EA), formality (FOR), and labour income (ING) consistently project toward the positive pole, whereas multidimensional poverty (PM), extreme multidimensional poverty (PM_ext), and income poverty (POB_ing, POB_ext) are located at the negative extreme. This persistence indicates that the territorial socioeconomic structure is robust and that the poverty-formality divide constitutes the dominant structural component in all periods.

Axis 2, although displaying greater interannual variability, preserves a coherent and interpretable pattern. Over the four years, underemployment (SUB) and the NEET population are consistently located on the positive pole, while the overall labour-force participation rate (TPG) and labour income (ING) concentrate on the negative pole. This confirms that the second axis captures labour-market heterogeneity and youth vulnerability, operating as a complementary yet persistent component of the factorial system.

At the territorial level, three stable spatial blocks emerge throughout 2021–2024. The Amazonian block – Napo, Orellana, Pastaza, and Morona Santiago – remains invariably at the negative extreme of Axis 1, aligned with poverty and deprivation vectors. Their annual contributions are among the highest in the factorial plane, consolidating their role as the structural core of national vulnerability.

The Andean-coastal block with higher labour well-being – Pichincha, Galápagos, Guayas, Azuay, and El Oro – appears consistently on the positive pole of Axis 1. This group maintains, year after year, a clear association with formality, higher incomes, and adequate employment. Galápagos and Pichincha stand out across the four biplots, systematically ranking as the best-positioned provinces in the structural labour-market space.

A third set, formed by provinces with intermediate patterns dependent on Axis 2 – Santa Elena, Manabí, Esmeraldas, and Los Ríos – is located each year toward the positive pole of the second axis. Their proximity to the underemployment and NEET vectors indicates persistent dynamics of labour precarity and youth disadvantage. Although this group exhibits slight variation across periods, its factorial behaviour follows the same interpretive pattern, suggesting that the youth-precarity component is a structural feature in these provinces.

In sum, the joint reading of the 2021–2024 biplots indicates that Ecuador’s territorial system exhibits remarkable stability in the way socioeconomic variables structure the labour space. The axes retain consistent meaning, territorial blocks recur without relevant change, and the dynamics of poverty, formality, and youth vulnerability are reproduced year after year. This reinforces the conclusion that regional gaps – particularly those separating the Amazonian provinces from Andean-coastal provinces with higher well-being – are not cyclical but structural and persistent.

### Partial triadic analysis

PTA was applied to the province × indicator × year array comprising four annual tables (2021–2024). The matrix of RV coefficients (Table 5) indicates a high degree of structural similarity across annual configurations. All coefficients are above 0.74, with the highest values observed between 2022 and 2023 (RV = 0.94) and between 2023 and 2024 (RV = 0.91). This suggests that, once measurement scales are controlled for, the employment-well-being geometry remains highly stable over the period. Similarity between 2021 and subsequent years is somewhat lower (RV between 0.75 and 0.82), consistent with the more transitional nature of the year immediately following the pandemic in Ecuador.

**Table 5:** RV coefficient matrix (vector correlation coefficients).

Year	A2021	A2022	A2023	A2024
**A2021**	1.0000	0.8197	0.7971	0.7462
**A2022**	0.8197	1.0000	0.9371	0.8891
**A2023**	0.7971	0.9371	1.0000	0.9060
**A2024**	0.7462	0.8891	0.9060	1.0000

**Fig 6:**
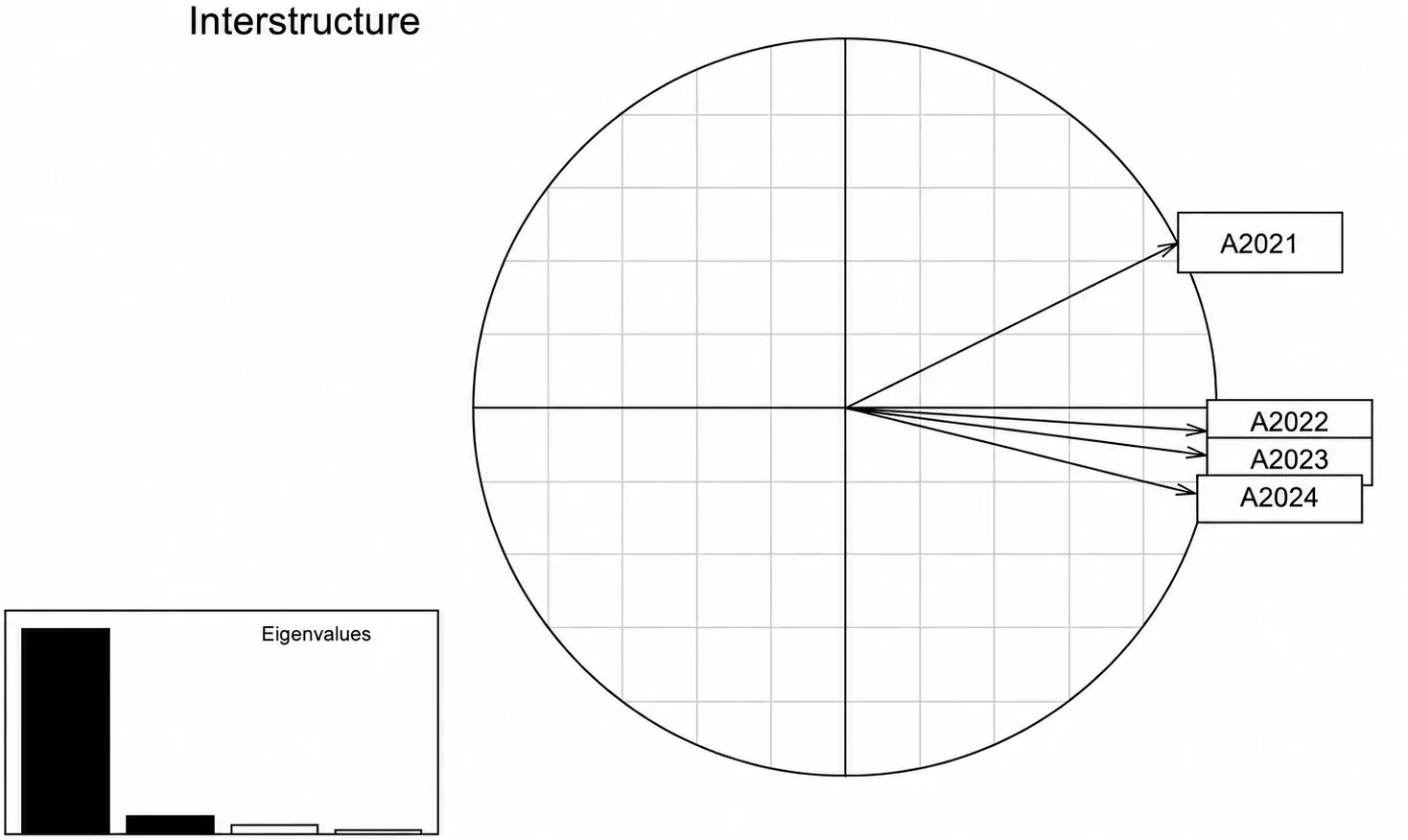
Interstructure map of the annual tables.

The interstructure analysis of the RV matrix shows that the first axis explains 88.8% of total inertia, while the second adds a further 7.0%; together, the first two components account for 95.8% of the between-table variation (Table 6). In the interstructure map (Fig 6), 2021 is projected slightly apart from the other years, whereas 2022, 2023, and 2024 cluster within the same quadrant, with very small angles between them. This configuration confirms that the multivariate employment-well-being structure consolidates from 2022 onwards, following the initial labour-market adjustment during the exit from the health crisis.

**Table 6:** Eigenvalues and explained inertia of the interstructure analysis.

Component	Eigenvalue	% Inertia	% Cumulative
**1**	3.552	88.8	88.8
**2**	0.280	7	95.8
**3**	0.108	2.7	98.5
**4**	0.061	1.5	100

**Fig 7:**
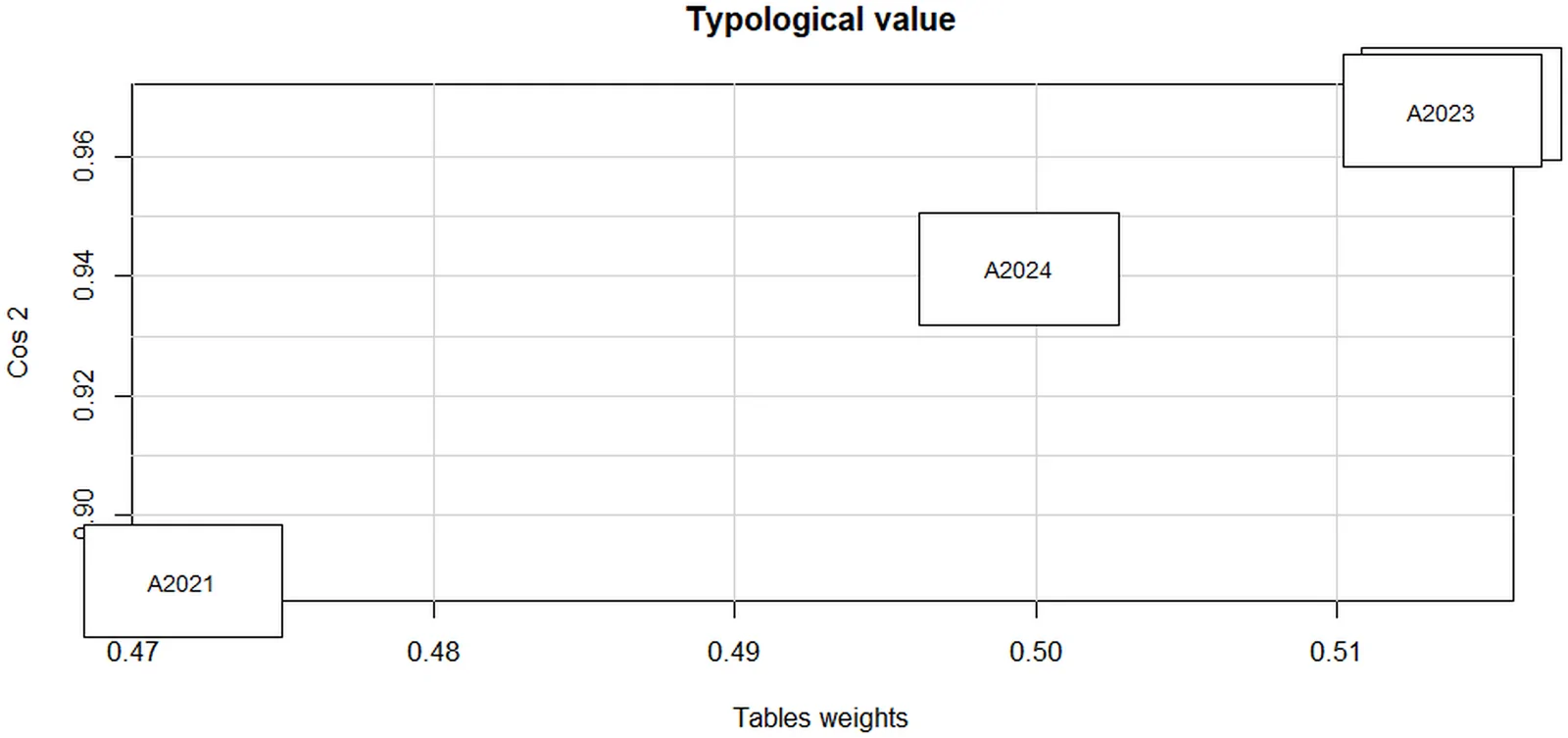
Typological values of annual tables in the compromise space.

Fig 7 summarises the typological values of the annual tables by combining each year’s weight in the compromise matrix (Table 7) with its squared cosine relative to the consensus space. The results indicate that all tables are very well represented in the compromise (cos^2^ > 0.88). Nevertheless, 2022 and 2023 exhibit the highest weights (0.514 and 0.514, respectively) and the best quality of representation (cos^2^ ≈ 0.97), confirming their role as the most typical years of the period’s employment-well-being pattern. Year 2021 shows the lowest weight and cos^2^(0.472 and 0.889), whereas 2024 occupies an intermediate position (weight ≈ 0.499; cos^2^≈ 0.941). Overall, the PTA suggests that the multivariate profile of Ecuador’s labour market in the post-pandemic stage stabilises around 2022–2023.

**Table 7:** K-table weights and squared cosines (cos^2^).

Year	K-table weight	cos²
**2021**	0.472	0.889
**2022**	0.514	0.969
**2023**	0.514	0.968
**2024**	0.499	0.941

The eigenvalue decomposition of the compromise matrix (Table 8) shows that the first axis explains 63.7% of total inertia and the second 16.9%, reaching a cumulative 80.6%; the third axis adds 10.5%, so that the first three components account for 91.0% of the total information (Table 8). This justifies interpreting results primarily in the (1–2) plane and, to a lesser extent, in the (1–3) space.

**Table 8:** Eigenvalues and explained inertia of the compromise matrix (PTA).

Component	Eigenvalue (λ)	% Inertia	% Cumulative
**1**	24.897	63.7	63.7
**2**	6.585	16.9	80.6
**3**	4.083	10.5	91.0
**4**	1.448	3.7	94.7
**5**	0.880	2.3	97.0
**6**	0.479	1.2	98.2
**7**	0.396	1.0	99.2
**8**	0.140	0.4	99.6
**9**	0.079	0.2	99.8
**10**	0.052	0.1	99.9
**11**	0.029	0.1	100.0

**Fig 8:**
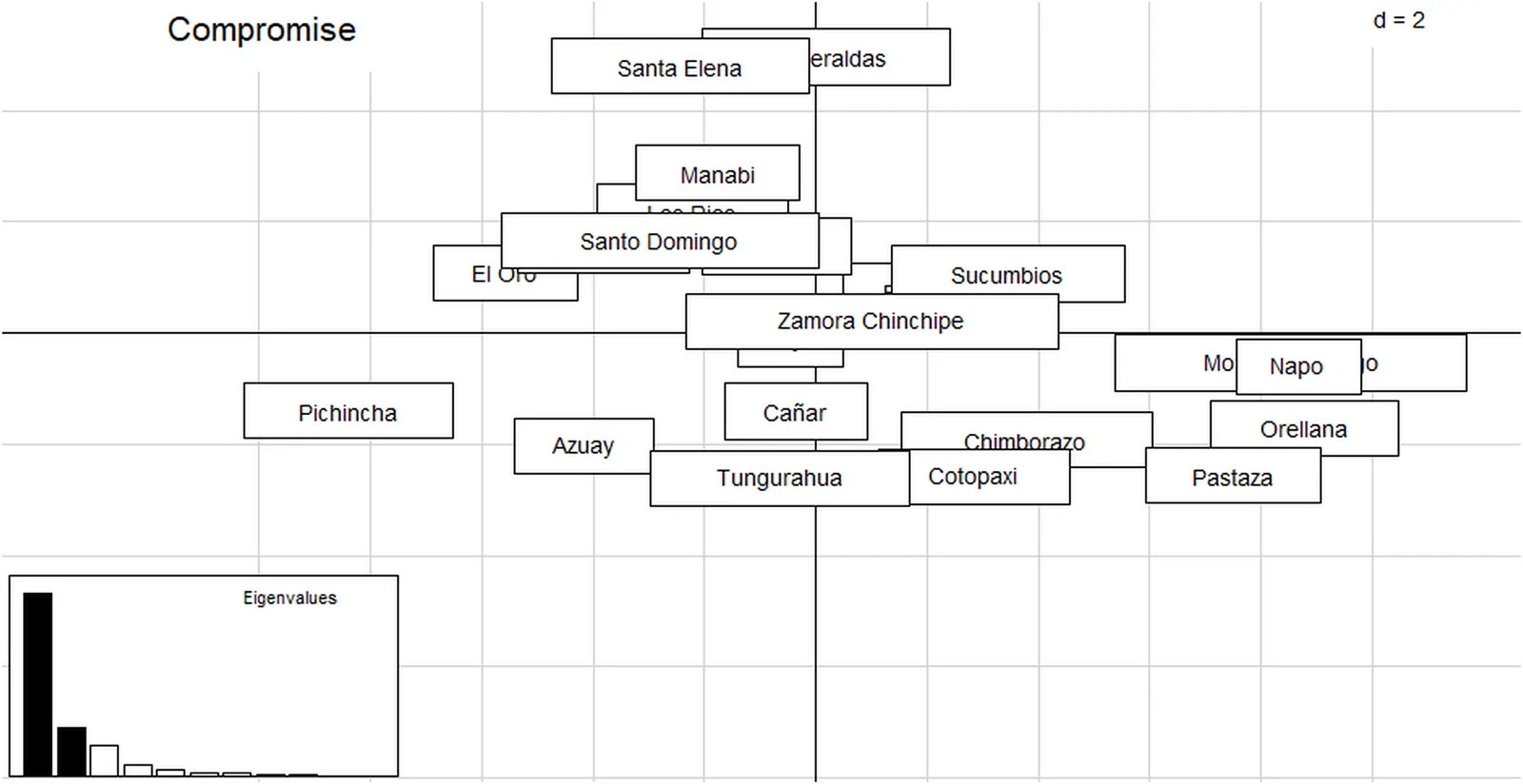
Compromise map of provinces (PTA).

In the compromise provinces biplot (Fig 8), Axis 1 reproduces the structural gradient identified in the annual analyses: on the right-hand side, the Amazonian block – Napo, Orellana, Pastaza, and Morona Santiago – together with Sucumbíos, is associated with higher levels of income and multidimensional poverty, lower formality, and poorer labour-market conditions. At the far left, Pichincha – and, to a lesser extent, Azuay and El Oro – are characterised by a more formal labour market and higher mean labour incomes. Provinces such as Manabí, Los Ríos, and Santo Domingo de los Tsáchilas cluster near the centre of the axis, reflecting intermediate positions along the employment-well-being gradient.

**Fig 9:**
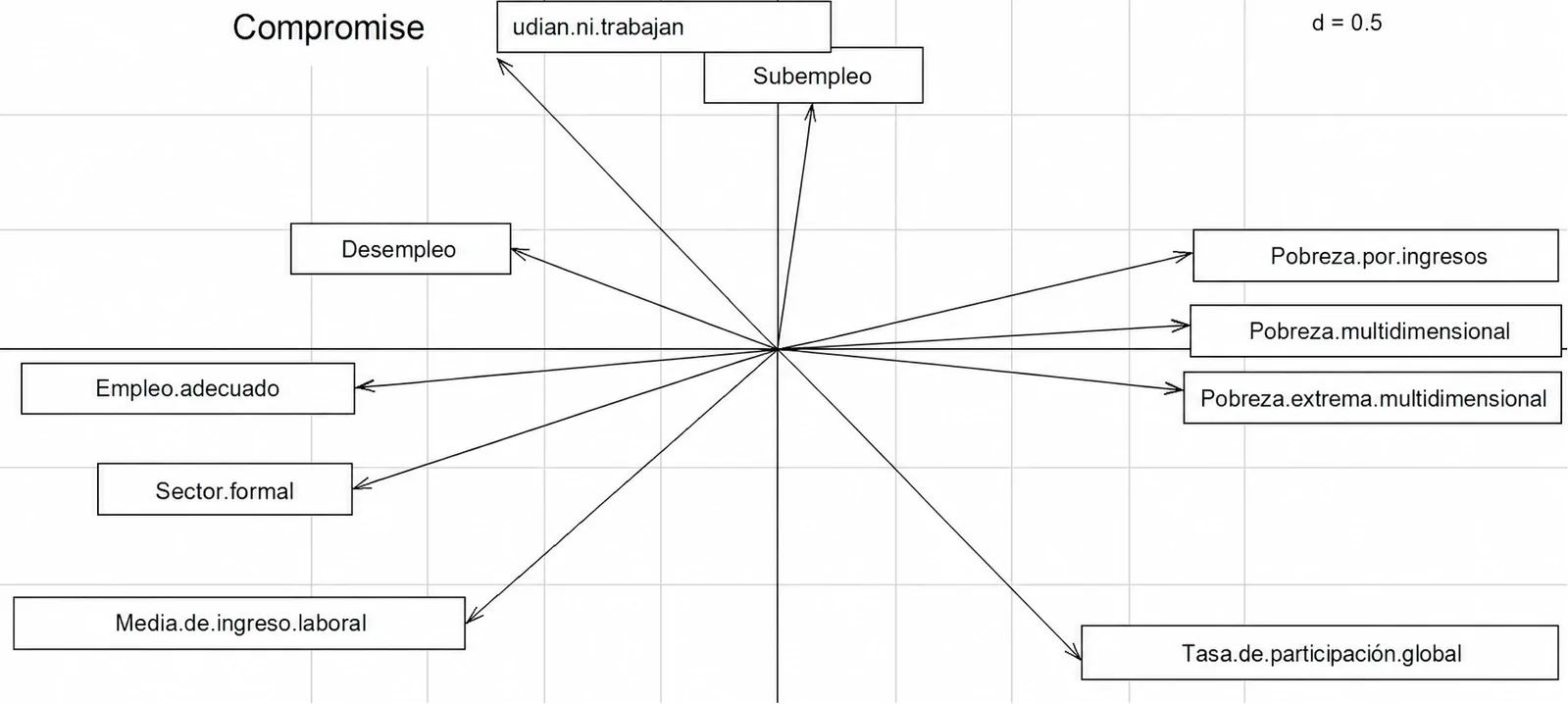
Compromise map of indicators (variables) (PTA).

The compromise indicators biplot (Fig 9) confirms the interpretation of the axes. Along Axis 1, the positive pole is defined by income poverty, extreme income poverty, and multidimensional poverty, whereas the negative pole groups adequate employment, formal employment, and mean labour income. The overall labour-force participation rate also aligns with the end associated with better labour outcomes. Axis 2 primarily reflects the contrast between underemployment and NEET youth on the upper side and lower levels of these forms of vulnerability on the lower side. Consequently, the consensus (1–2) plane summarises a main poverty-formality axis and a secondary axis capturing youth labour vulnerability and underemployment, in line with the annual HJ-biplots.

Overall, PTA indicates that the employment-well-being structure of Ecuador’s provinces is remarkably stable over 2021–2024, with a slight singularity in 2021 and consolidation of the pattern from 2022 onwards. The compromise summarises this behaviour in a factorial space that clearly distinguishes provinces characterised by high multidimensional poverty and low formality from territories with higher incomes and adequate employment, while a secondary axis captures the intensity of underemployment and youth exclusion. This common structure provides the basis for analysing province-specific trajectories in the consensus plane.

## Discussion

### Temporal stability of the employment-well-being structure (RV matrix and interstructure)

The PTA results show that the four annual employment-well-being tables (2021–2024) are highly coherent with one another. The first eigenvalue of the interstructure accounts for close to 89% of total inertia and the second adds around 7%, so that the first two components explain more than 95% of the between-table variability. This dominance of the first axis indicates the presence of a very strong common pattern in the relationship between employment, poverty, and income over the period analysed.

The interannual RV coefficients reinforce this interpretation: all vector correlations exceed 0.74, with particularly high values between 2022 and 2023 (RV ≈ 0.94) and between 2023 and 2024 (RV ≈ 0.91). Substantively, this suggests that, once the initial post-pandemic adjustment in 2021 had passed, the multivariate geometry of Ecuador’s labour market stabilised rapidly around a relatively robust pattern. This result is consistent with regional evidence documenting an employment recovery in Latin America driven by lower-productivity and informal occupations, alongside a structure of inequalities that remains highly persistent over time [47,63].

In the interstructure map, 2021 appears slightly separated from the other years, whereas 2022, 2023, and 2024 cluster within the same quadrant with very small angles between them. This supports the interpretation of 2021 as a transitional year between the pandemic shock and the new labour-market normal. Recent studies report similar dynamics in other Latin American countries: an abrupt employment decline during 2020–2021, followed by a reallocation toward informal and lower-quality sectors that consolidates a new precarious equilibrium [53,54].

### Consensus geometry and territorial profiles (compromise matrix)

The eigenvalue decomposition of the compromise matrix shows that the first axis explains around 64% of total inertia and the second close to 17%, reaching roughly 81% cumulatively; the third component adds just over 10%, so that the first three axes concentrate approximately 91% of the information. This highly concentrated structure supports a confident interpretation of the (1–2) plane as a synthetic representation of the provincial employment-well-being system.

In the variable compromise, Axis 1 organises a very clear gradient between provinces with stronger labour-market integration and higher incomes – positively associated with adequate employment, formality, and mean labour income – and territories characterised by high levels of income poverty, multidimensional poverty, and their extreme variants. This type of opposition between ‘dynamic cores’ and ‘lagging territories’ has been widely documented in the literature on regional inequalities in Latin America [53,55].

Axis 2 introduces a different nuance, mainly linked to the combination of underemployment and youth vulnerability (NEET ages 15–24) versus relatively more stable employment configurations. Provinces with high underemployment and large shares of young people not in education, employment, or training are located on the positive pole of this axis, whereas those with lower NEET levels tend to occupy intermediate or negative positions. This association is consistent with international evidence highlighting the link between precarious early-career trajectories and persistent risks of social exclusion [50].

In the province compromise, the (1–2) plane clearly reproduces the territorial segmentation observed in the annual HJ-biplots:

- An Andean and coastal block – Pichincha, Azuay, El Oro, Guayas, and, in a distinctive way, Galápagos – lies on the pole of higher labour well-being, with high levels of adequate employment, formality, and income.
- At the opposite extreme, the Amazonian provinces (Napo, Pastaza, Morona Santiago, Orellana, and Sucumbíos) are concentrated in the quadrant of greatest precarity, combining high rates of multidimensional poverty, extreme poverty, and underemployment.
- An intermediate group of provinces (Manabí, Los Ríos, Santo Domingo, Zamora Chinchipe, among others) is distributed around the centre, reflecting mixed conditions and internal heterogeneity.

Table 9 reports descriptive statistics for the compromise coordinates and summarises the distribution of provincial positions in the consensus space. These results are consistent with previous diagnoses identifying territorial pockets of poverty and labour precarity in rural and Amazonian areas, where deficits in basic services, limited productive diversification, and scarce formal employment tend to converge [55].

**Table 9:** Descriptive statistics of the compromise coordinates.

Year	Axis	Mean	Std. Dev.	Min	Max	Skew	Kurtosis
2021	Axis1	0	2.41338	−4.63	4.99	−0.008	−0.006
	Axis2	0	1.33456	−2.65	2.92	−0.050	−0.011
2022	Axis1	0	2.60279	−4.69	5.19	−0.307	−0.126
	Axis2	0	1.45558	−4.40	2.36	−0.953	2.397
2023	Axis1	0	2.58817	−4.99	5.25	−0.274	−0.088
	Axis2	0	1.35276	−3.78	2.64	−0.430	1.718
2024	Axis1	0	2.59631	−5.63	5.45	−0.133	0.072
	Axis2	0	1.37980	−3.60	2.62	−0.259	0.782

### Typical years and stability of the post-pandemic pattern

The typological values of the annual tables – combining each year’s weight in the compromise (Table 7) and its cos^2^ relative to the consensus space – show that all matrices are very well represented (cos^2^> 0.88). However, 2022 and 2023 stand out as the most typical years: they display the highest weights (≈ 0.51) and the best quality of representation (cos^2^ ≈ 0.97). By contrast, 2021 has the lowest weight and cos^2^while 2024 occupies an intermediate position.

This configuration suggests that the employment-well-being structure emerging after the pandemic’s initial shock consolidated in 2022−2023, stabilising territorial contrasts between provinces with higher and lower job quality. This is consistent with evidence indicating that, in the region, the post-COVID-19 recovery relied largely on low-productivity and highly informal sectors, thereby consolidating a new labour-market equilibrium with persistent gaps [52,63].

### Provincial trajectories in the consensus space

**Fig 10:**
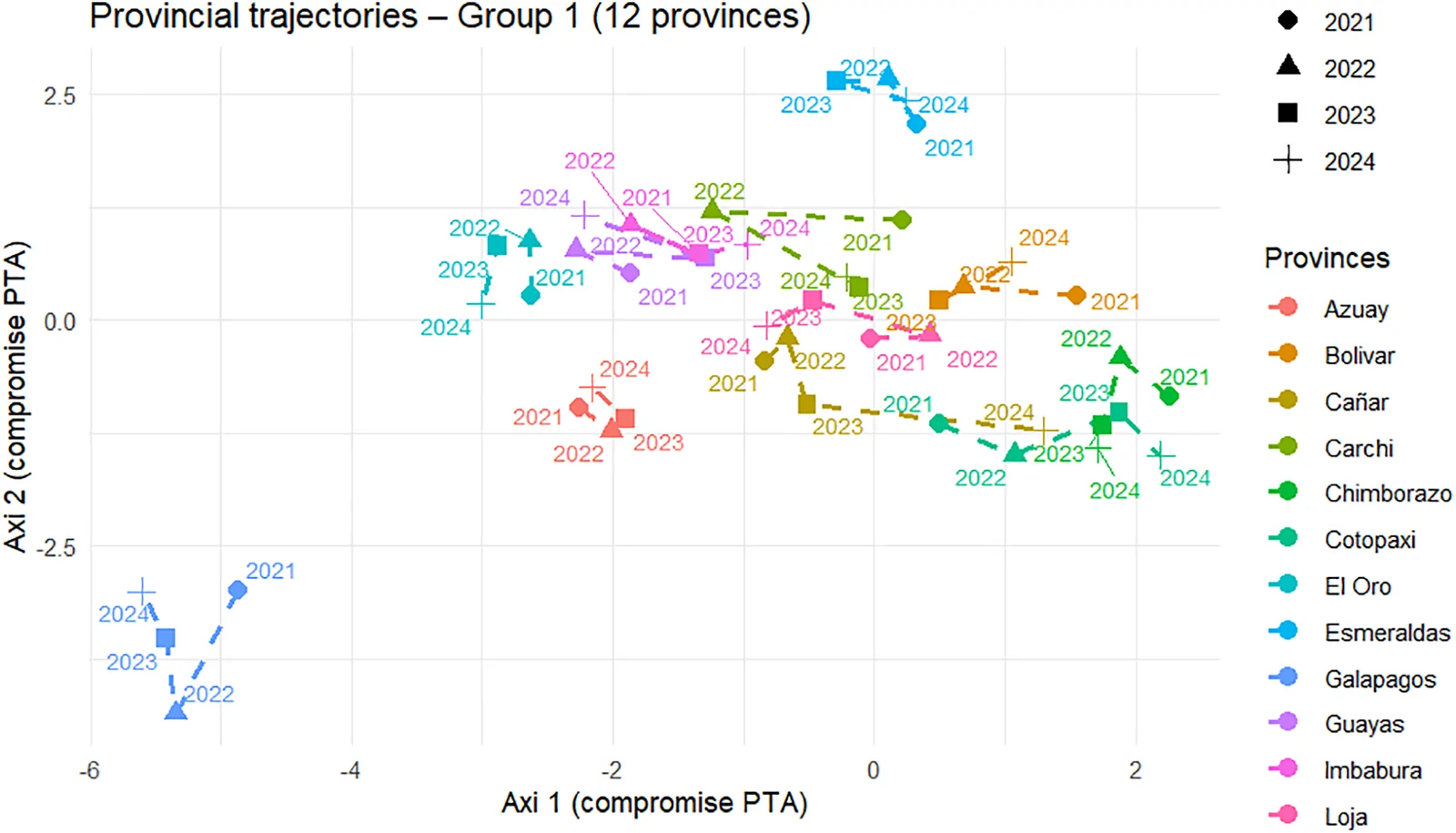
Provincial trajectories (Azuay – Bolívar – Cañar – Carchi – Chimborazo – Cotopaxi. El Oro – Esmeraldas – Galápagos – Guayas – Imbabura – Loja).

**Fig 11:**
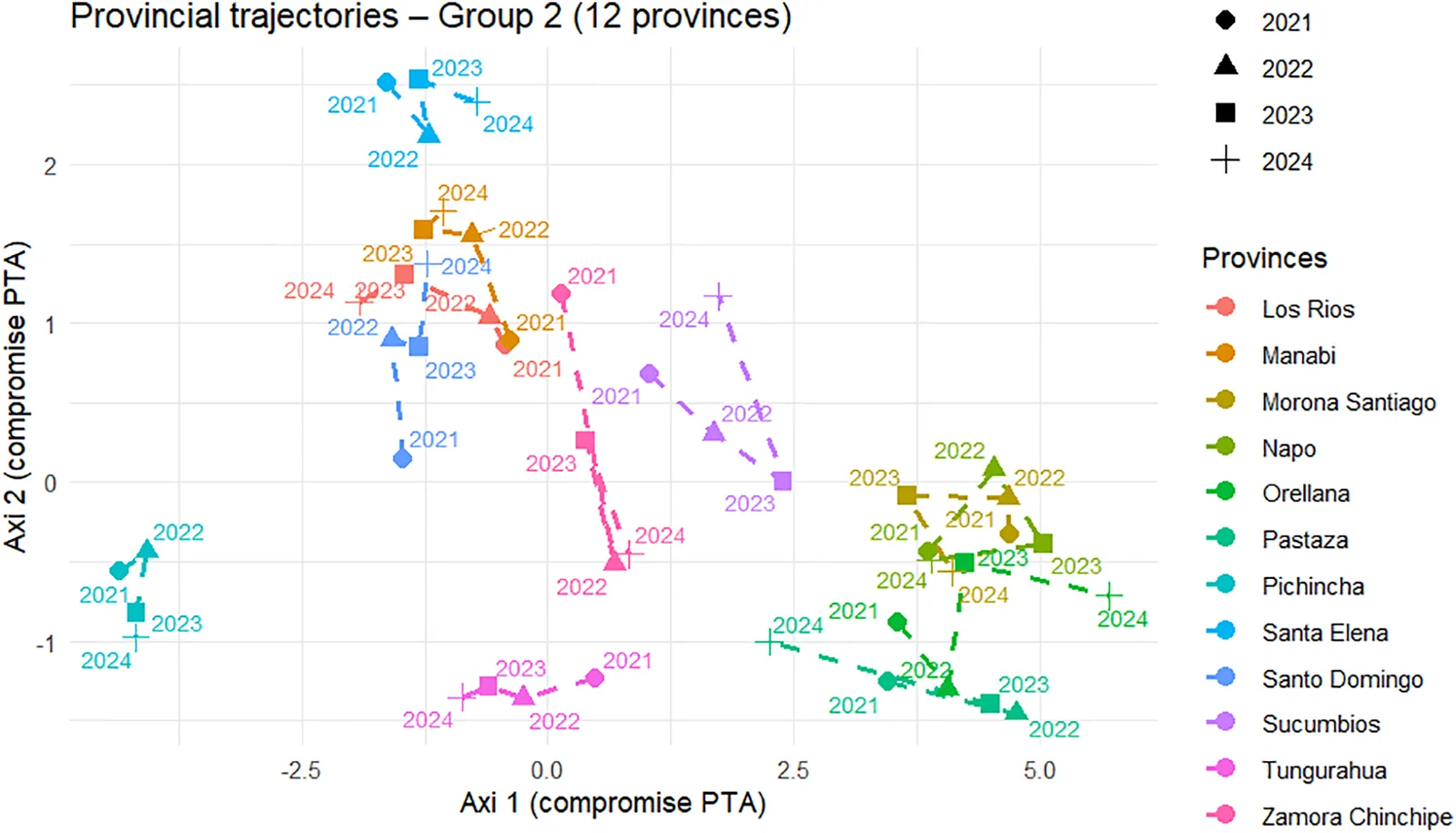
Provincial trajectories (Los Ríos – Manabí – Morona Santiago – Napo – Orellana – Pastaza. Pichincha – Santa Elena – Santo Domingo – Sucumbíos – Tungurahua – Zamora Chinchipe).

Projecting each annual table onto the compromise matrix makes it possible to reconstruct provincial trajectories in the employment-well-being space over 2021–2024. Although movements are generally moderate – consistent with the high similarity across years – three qualitative patterns can be identified. Figs 10 and 11 display these trajectories for the full set of provinces.

1. **High and stable well-being provinces.**

Pichincha, Azuay, Galápagos and, to a lesser extent, El Oro maintain consolidated positions on the pole associated with higher adequate employment, formality, and income, exhibiting short trajectories and limited oscillations. This indicates that the post-pandemic recovery did not substantially alter their relative advantage, in line with studies documenting greater resilience in diversified regional economies with a high concentration of formal employment [53].

2. **Territories of persistent precarity.**

The Amazonian provinces show trajectories that remain in the quadrant characterised by higher multidimensional poverty and underemployment, with no meaningful shifts toward the centre of the plane. This persistence reinforces the notion of ‘territories of vulnerability’ where labour and well-being disadvantages overlap, consistent with evidence for other peripheral regions in Latin America [55].

3. **Provinces in transition or reconfiguration.**

An intermediate group – for example, Manabí, Los Ríos, Santo Domingo de los Tsáchilas, and Zamora Chinchipe – shows trajectories moving closer to the centre of the consensus space, which may be interpreted as internal recomposition processes involving adequate employment, informality, and poverty. PTA does not identify causality, but it provides a useful map for targeting more detailed national analyses, for instance through panel models or sectoral case studies.

### NEET youth and structural vulnerability

Taken together, the annual HJ-biplots allow for a more nuanced interpretation of the NEET phenomenon in Ecuador’s labour market. The results show that the NEET (15–24) variable does not align with the income-poverty or multidimensional-poverty vectors; instead, together with unemployment and underemployment, it defines an almost independent axis of labour vulnerability. Across the four years analysed, the NEET vector remains in the upper half-plane of the factorial plane and lies close to the unemployment and underemployment vectors, while being clearly separated from the poverty-related block of variables. This configuration suggests that youth inactivity is more closely associated with difficulties of labour-market insertion and with the quality of available jobs than with household income insufficiency per se.

This evidence speaks directly to the international literature linking NEET status to segmented labour markets, low-quality employment, and disrupted educational trajectories rather than to monetary poverty in a strict sense [50]. In the Latin American context, the findings are consistent with studies documenting higher probabilities of being NEET in territories with limited formal-employment opportunities and heterogeneous educational quality [44,64], while adding an important territorial nuance: the problem is not concentrated exclusively in the structurally poorest provinces.

Axis 1 of the HJ-biplots summarises a highly stable territorial gradient of labour well-being: provinces with higher mean labour income, adequate employment, and formality lie on the right-hand side, whereas provinces with higher poverty rates and greater participation in low-quality occupations lie on the left-hand side. Within this gradient, the projection of NEET into the upper-right quadrant in most years suggests that the highest shares of young people who are not in education, employment, or training tend to be located in ‘intermediate-performance’ provinces, characterised by more segmented labour markets and particularly fragile school-to-work transitions.

**Fig 12:**
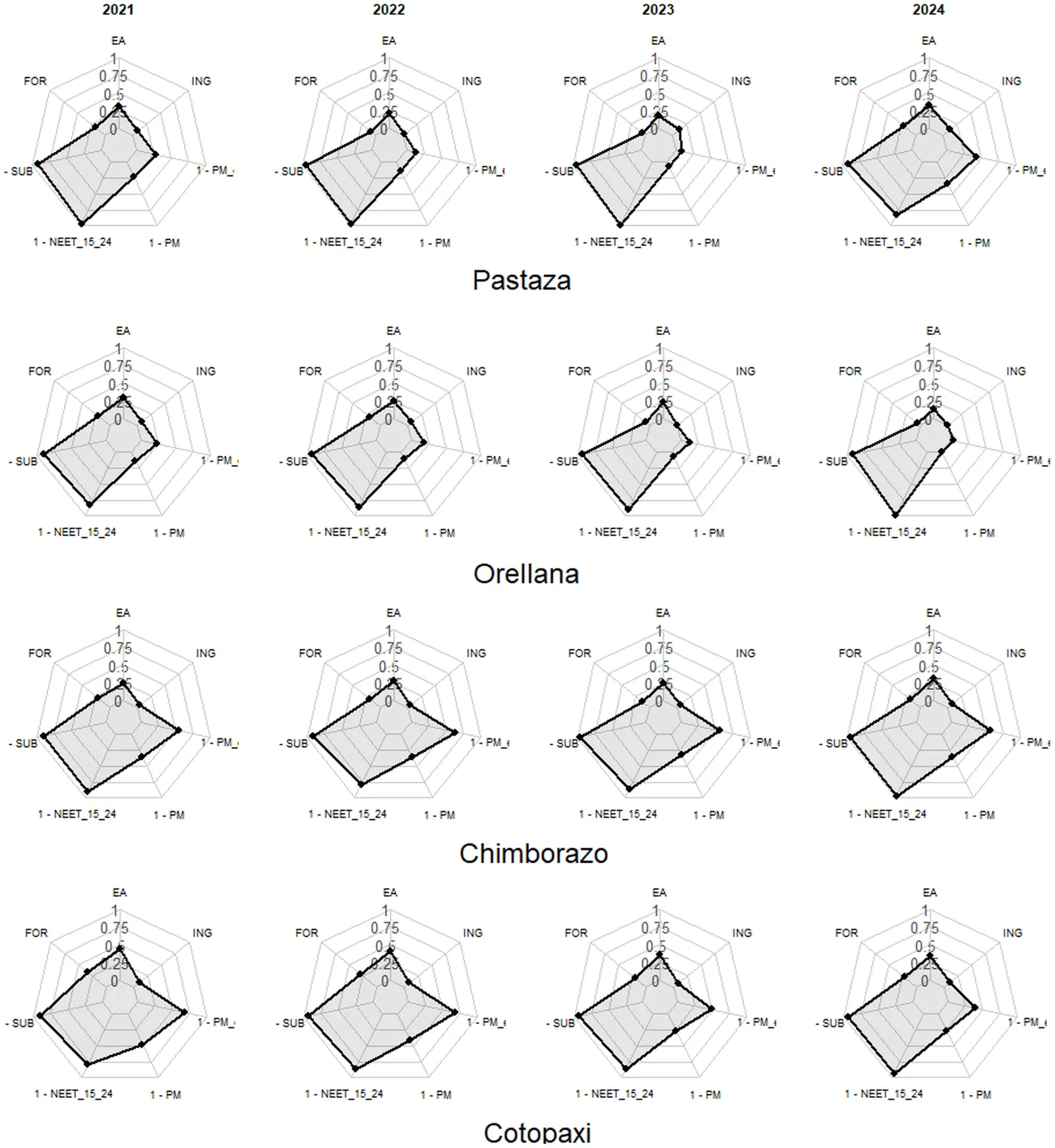
Employment–well-being radar chart (high-vulnerability provinces).

**Fig 13:**
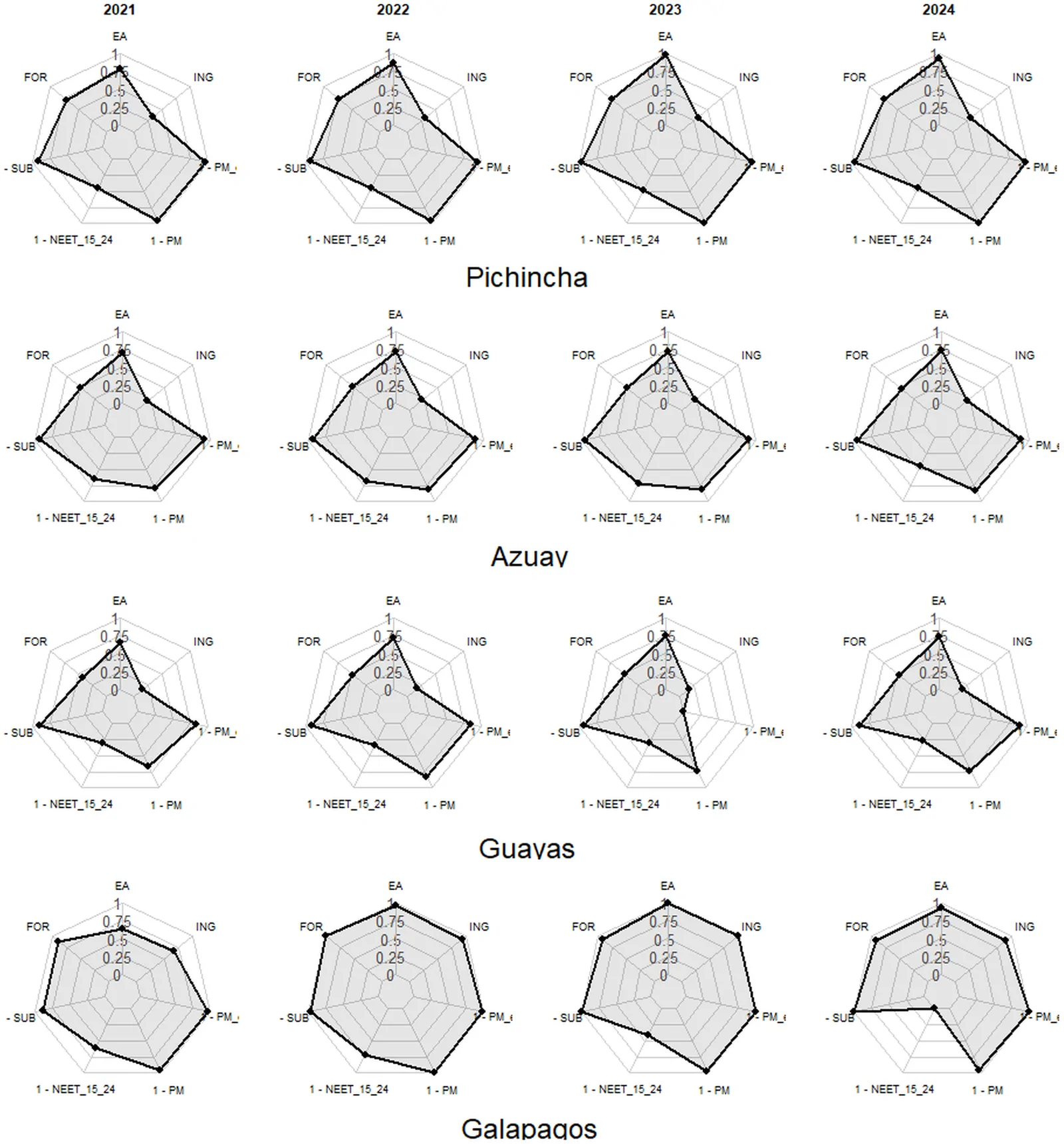
Employment–well-being radar chart (low-vulnerability provinces).

The compromise map of variables reinforces this interpretation by showing that income poverty, multidimensional poverty, and extreme multidimensional poverty form a compact block along the material-deprivation axis, whereas NEET, unemployment, and underemployment cluster in a distinct direction, defining a specific dimension of disadvantage associated with youth labour-market insertion. From a public-policy perspective, this implies that interventions targeting NEET youth cannot be limited to broad poverty-reduction strategies; rather, they require territorial approaches that integrate improvements in educational quality, vocational guidance, school-to-work bridges, and labour-intermediation mechanisms tailored to local productive structures. This contrast is also visible in the employment-well-being radar profiles of representative high- and low-vulnerability provinces shown in Figs 12 and 13.

### Territorial blocks and contributions in relation to the existing literature

In comparative terms, the three territorial blocks identified here speak directly to prior evidence on structural heterogeneity in Latin America and, in particular, Ecuador. The high-vulnerability Amazonian block confirms the existence of territories trapped in persistent combinations of multidimensional poverty, informality, and low employment productivity, in line with diagnoses of deep territorial gaps in the Andean region [54] and evidence on spatial inequalities in income and deprivations in Ecuador [12,46,65]. However, the employment-well-being framework proposed here shows that these gaps are not limited to income; rather, they are sustained by labour-market configurations in which underemployment and informality constitute the core of disadvantage.

The Andean-coastal block with higher labour well-being aligns with the literature documenting territorial ‘winners’ in Latin America’s inequality-reduction process and expansion of the middle class [7,8,53]. Nevertheless, the HJ-biplot indicates that this advantage is not explained solely by higher income levels, but by a combination of adequate employment, formality, and lower exposure to multidimensional poverty, which is consistent with the job-quality approach (QoE) and with multidimensional employment indices proposed for the region [47,49,63]. The third set of provinces, with intermediate profiles and more unstable trajectories, adds an important nuance: these are not merely transition zones toward well-being, but territories where job quality and youth vulnerability can deteriorate without necessarily implying a collapse in average income.

Regarding NEET youth, the results reinforce international evidence documenting the multidimensional nature of this condition [1,25,27,50]. The fact that NEET (15–24) defines an almost independent axis in the consensus space – associated with unemployment and underemployment but only partially with poverty measures – suggests that in Ecuador, as in other countries in the region, there is a youth segment trapped at the intersection of segmented labour markets and education systems with limited pathways into decent work. This finding complements analyses of informality, social protection, and labour vulnerability in Latin America [18,51] by adding an explicitly territorial dimension to the discussion.

In this context, the three-way approach applied here – provinces × indicators × years – adds insight that studies relying exclusively on inequality regressions do not capture with the same clarity. While econometric models are well suited to identifying marginal effects and testing causal hypotheses, the combined use of HJ-biplots and PTA makes the geometry of the employment-well-being system visible: how provinces and indicators are jointly organised in factorial space, how stable this structure is over time, and what trajectories territories trace in the consensus space. This geometric perspective is particularly useful in settings with high collinearity among variables, small subnational sample sizes, and strong interest in comparative spatial patterns. It should therefore be viewed as a complement to, rather than a substitute for, traditional econometric approaches.

### Methodological contributions and future agenda

From a methodological perspective, combining annual HJ-biplots with PTA and applying them to official household survey data offers several advantages over more traditional approaches based solely on regressions or univariate indicators. First, it enables the simultaneous representation of provinces and indicators in low-dimensional spaces, facilitating an immediate reading of correlations among variables and territorial similarities [57]. Second, PTA provides a formal framework for decomposing total variability into interstructure, compromise, and intrastructure components, synthesising information from multiple time slices into a stable consensus matrix [35,60].

These methods have proven useful in diverse fields, including the analysis of gender inequalities in the SDGs, environmental quality, and financial-system assessment [60,61]. To the best of our knowledge, applying them to the employment-well-being relationship at the provincial level in Ecuador constitutes a novel contribution to the literature and opens the door to future extensions: incorporating additional years, introducing urban-rural disaggregation, linking the framework to sectoral indicators, or integrating it with econometric models that use PTA-extracted factors as explanatory variables.

Beyond its exploratory nature, the combined use of HJ-biplots and Partial Triadic Analysis establishes an operational framework for territorial monitoring of the employment-well-being system. Annual HJ-biplots jointly represent, in a single plane, the articulation between employment quantity and quality, monetary and multidimensional poverty, and youth inactivity, offering a compact reading of provincial structure each year. PTA adds an explicit temporal dimension by extracting a compromise matrix that summarises the common structure of 2021–2024 and by tracing provincial trajectories in the consensus space. Taken together, this multivariate geometric approach does not merely characterise a static snapshot; it provides a replicable instrument for tracking the evolution of territorial gaps and detecting persistent patterns of labour exclusion over time.

This potential is particularly relevant in the context of the Sustainable Development Goals. The study’s indicators connect directly to SDG 1 (No Poverty) through income poverty and multidimensional poverty measures, and to SDG 8 (Decent Work and Economic Growth) through adequate employment, informality, underemployment, and youth NEET variables. By integrating these dimensions into a single geometric space and incorporating time, the HJ-biplot-PTA approach provides a technical basis for territorial monitoring dashboards that would enable governments to assess provincial convergence or divergence periodically with respect to SDG 1 and SDG 8 targets. This creates scope for incorporating these methods into official statistical and planning systems – for example, as an analytical complement to standard ENEMDU indicators – and for using provincial trajectories in the compromise space as an input for evaluating employment, social protection, and poverty-reduction policies at the subnational level.

From a public-policy standpoint, the evidence suggests that the post-pandemic recovery agenda should focus not only on the number of jobs created, but also on their multidimensional quality and on the reduction of persistent territorial gaps. Identifying provincial blocks with clearly differentiated profiles provides an empirical basis for designing territorial employment and well-being strategies that integrate labour, social, and productive-development policies.

A first area for action concerns comprehensive territorial policies for the Amazon region, where the compromise shows trajectories persistently located at the pole of higher multidimensional poverty, underemployment, and low labour incomes. In these territories, isolated training or entrepreneurship programmes are insufficient; rather, combined packages are needed that integrate: (i) investment in basic infrastructure and connectivity to reduce access costs to formal jobs; (ii) fiscal and financial incentives to attract higher-productivity activities that generate adequate employment; and (iii) the expansion of social protection floors and contributory coverage for workers currently concentrated in informal segments and subsistence agriculture. Without such an integrated strategy, Amazonian trajectories are likely to reproduce a ‘territorial trap’ pattern in which cyclical improvements do not translate into structural changes in the employment-well-being profile.

Second, the results suggest that NEET youth in intermediate-profile provinces – neither the best nor the worst along the labour well-being gradient – require a tailored response. The fact that NEET (15–24) forms its own axis, associated with unemployment and underemployment but only partially with poverty, indicates that many young people become trapped in a grey zone: territories with mid-level income and formality but weak bridges between the education system and the labour market. For these contexts, the evidence supports prioritising policies such as dual technical training linked to local sectors, temporary incentives for formal youth hiring, province-scale career guidance and labour-intermediation services, and second-chance education pathways to re-engage those who have left school.

Finally, the identification of relatively stable provincial blocks opens the door to a more refined typology of interventions. Rather than uniform nationwide policies, the results support the construction of at least three families of strategies: (i) a ‘closing structural gaps’ agenda for the Amazon region and other high-vulnerability provinces; (ii) a ‘consolidation and job-quality’ agenda for high-well-being provinces, focused on productivity, innovation, and the reduction of residual informal segments; and (iii) a ‘preventing new vulnerability clusters’ agenda for intermediate provinces, with an explicit focus on NEET youth and improving job quality in services and commerce. If policymakers in Ecuador use this employment-well-being map as an input, the main change is not only diagnostic; it is also the ability to prioritise territories, target populations, and policy mixes far more precisely than with isolated traditional indicators.

### Limitations

Several limitations should be acknowledged. First, the analysis is based on 24 provincial units, which is adequate for descriptive multivariate mapping but limits stronger claims about the statistical stability of very small differences in coordinates or contributions. Second, provincial averages may hide substantial intraprovincial heterogeneity, especially urban-rural contrasts in provinces such as Pichincha or Guayas, where favourable labour indicators in metropolitan areas can coexist with severe multidimensional deprivation in peripheral zones.

Third, the study is exploratory and descriptive: HJ-biplots and PTA summarise multivariate structure and temporal trajectories, but they do not identify causal mechanisms. Future research should extend the time horizon, introduce urban-rural and gender disaggregation, and complement the geometric approach with panel or spatial econometric models.

## Conclusion

The central objective of this study was to analyse the structure and territorial evolution of employment-well-being inequalities across Ecuador’s 24 provinces between 2021 and 2024, integrating indicators of employment quantity and quality, monetary and multidimensional poverty, and NEET youth inactivity. By combining annual HJ-biplots and Partial Triadic Analysis (PTA), we proposed a multivariate geometric framework that enables the simultaneous representation of provinces and indicators, the synthesis of temporal information, and the tracing of territorial trajectories within a consensus space.

Regarding the first objective, the results show that the provincial employment-well-being system is organised primarily by a gradient dominated by job quality and labour income rather than by unemployment. In every year analysed, a main axis emerges that contrasts provinces with higher adequate employment, greater formality, and higher incomes against territories where underemployment, informality, and deprivations predominate. Income poverty and multidimensional poverty measures align systematically with this gradient, confirming the close articulation between labour-market disadvantages and multiple deprivations.

With respect to the second objective, the territorial distribution reveals three relatively stable blocks. An Amazonian block persistently occupies the pole of highest vulnerability, combining elevated multidimensional poverty, underemployment, and low incomes. An Andean-coastal block, including provinces with more dynamic labour markets such as Pichincha and Galápagos, concentrates the most favourable employment-well-being profiles. A third set of provinces displays intermediate configurations and more unstable trajectories, where small shifts in job quality and informality may translate into meaningful advances or setbacks. This pattern confirms the existence of structural territorial gaps consistent with the regional literature, while adding an integrated employment-well-being reading that goes beyond income alone.

Regarding the third objective, PTA reveals high similarity across annual structures and a compromise matrix that synthesises a relatively stable post-pandemic ‘average’ profile. Trajectories in the consensus space suggest slow adjustment processes, with no clear convergence across provinces: some improve slightly in relative terms, others stagnate or deteriorate, but no structural shifts are observed that would alter the hierarchy of territorial blocks. This reinforces the view that employment-well-being gaps in Ecuador have a strongly structural component that is difficult to reverse in the short run.

A specific finding, linked to the fourth objective and the youth dimension, is that NEET (15–24) defines its own vulnerability axis that is not reducible to poverty. In both the compromise and the annual HJ-biplots, NEET is consistently associated with unemployment and underemployment, but only partially with monetary and multidimensional poverty measures. This suggests the existence of a segment of young people trapped in a ‘grey zone’: territories with medium income and intermediate formality where, nonetheless, pathways between the education system and the labour market are weak. This evidence supports the need for targeted policies for NEET youth in intermediate provinces, beyond approaches focused exclusively on poor households.

From a methodological standpoint, the study shows that combining HJ-biplots and PTA constitutes an applied framework for territorially monitoring employment-well-being inequalities using official microdata from ENEMDU and INEC. This approach integrates, within a single analytical device, employment quantity and quality, monetary and multidimensional poverty, and youth inactivity, generating comparable maps by province and year and a consensus space in which trajectories can be tracked. In this sense, it offers a replicable tool that can be incorporated into statistical and planning systems to monitor SDG 1 and SDG 8 territorially and to evaluate employment and poverty-reduction strategies at the subnational level.

Finally, the study opens a future research agenda. Potential extensions include incorporating longer time series to capture full economic cycles; urban-rural and gender disaggregation to analyse within-province gaps; integrating sectoral or productivity indicators to refine the interpretation of territorial profiles; and combining the geometric approach with econometric models that use PTA-extracted factors as explanatory variables for specific outcomes (e.g., social mobility, youth job quality, or educational performance). Despite these limitations, the results show that the HJ-biplot-PTA approach provides a valuable complement to conventional analyses and offers a solid basis for designing employment and well-being policies with a territorial and generational focus in Ecuador and, potentially, in other Latin American countries.

The dataset supporting the findings of this study is publicly available in Zenodo [66].

## Supporting information

S1 File Dataset. Provincial dataset used in the HJ-biplot and PTA analyses (Ecuador, 2021–2024).
(XLSX)

S2 File Codebook. Variable dictionary and operational definitions of the indicators included in the analyses.
(DOCX)

S3 File Codebook variables. (CSV)

S4 File README. (DOCX)
