# Supplementary material for: Mapping territorial labour-related well-being inequalities in Ecuador: A multivariate geometric approach
Source: PLoS One. 2026 Aug 31;21(8):e0343199. doi: 10.1371/journal.pone.0343199 (PMC13529017; doi:10.1371/journal.pone.0343199)
Supplement: Supplementary file 1 [file pone.0343199.s002.docx]

**S2 Codebook — Variable Dictionary**

Minimal dataset for PLOS ONE submission PONE-D-26-05632R1

Manuscript title: Mapping Territorial Labour-Related Well-Being Inequalities in Ecuador: A Multivariate Geometric Approach

Author: Bill Jonathan Serrano Orellana

Dataset file: S1 Dataset.xlsx

Coverage: Ecuadorian provinces, 2021-2024

Unit of analysis: Province-year

Note: Rate variables are coded as proportions between 0 and 1. Multiply by 100 to express them as percentages. Average labour income is expressed in current USD. Population variables are counts of persons.

# Variable dictionary

| **Dataset field** | **Short name** | **Full name** | **Definition** | **Type** | **Unit / scale** | **Analytical role** | **Notes** |
| --- | --- | --- | --- | --- | --- | --- | --- |
| Provincia | PROV | Province | Ecuadorian province used as the territorial unit of analysis. | Categorical | Province name | Identifier | There are 24 provinces in each annual sheet. |
| Tasa de participación bruta | TPB | Crude labour force participation rate | Share of the total population that belongs to the economically active population or labour force. | Numeric | Proportion (0–1) | Contextual labour indicator | Multiply by 100 to express as percentage. |
| Tasa de participación global | TPG | Overall labour force participation rate | Percentage of the working-age population that is employed or actively seeking employment. | Numeric | Proportion (0–1) | Main analytical indicator | Variable abbreviation used in the manuscript: TPG. |
| Desempleo | DES | Unemployment rate | Percentage of the labour force that is without work, available for work, and actively seeking employment. | Numeric | Proportion (0–1) | Main analytical indicator | Variable abbreviation used in the manuscript: DES. |
| Empleo | EMP | Employment rate | Share of the labour force that is employed. | Numeric | Proportion (0–1) | Contextual labour indicator | Complementary labour-market indicator. |
| Empleo adecuado | EA | Adequate employment rate | Employed individuals with earnings equal to or above the minimum wage and working at least 40 hours per week; or working fewer than 40 hours per week with no desire and no availability to work additional hours. | Numeric | Proportion (0–1) | Main analytical indicator | Variable abbreviation used in the manuscript: EA. |
| Subempleo | SUB | Underemployment rate | Employed individuals with earnings below the minimum wage and/or working fewer hours than desired, who are available to work additional hours. | Numeric | Proportion (0–1) | Main analytical indicator | Variable abbreviation used in the manuscript: SUB. |
| Empleo no remunerado | ENR | Unpaid employment rate | Share of employed individuals who work without receiving monetary remuneration. | Numeric | Proportion (0–1) | Contextual labour indicator | Included in the dataset as an additional employment-composition variable. |
| Otro empleo no pleno | OENP | Other non-full employment rate | Share of employed individuals classified in other forms of non-full employment, excluding adequate employment, underemployment, and unpaid employment categories. | Numeric | Proportion (0–1) | Contextual labour indicator | Included in the dataset as an additional employment-composition variable. |
| Sector formal | FOR | Formal employment share | Percentage of employed workers in establishments registered with a tax identification number (RUC), used as a proxy for formality. | Numeric | Proportion (0–1) | Main analytical indicator | Variable abbreviation used in the manuscript: FOR. |
| Sector informal | INF | Informal employment share | Percentage of employed workers in establishments not registered with a tax identification number (RUC), used as a proxy for informality. | Numeric | Proportion (0–1) | Contextual labour indicator | Complementary indicator to the formal employment share. |
| Personas que no estudian ni trabajan | NEET_15_24 | NEET rate (ages 15–24) | Percentage of young people aged 15–24 who are not in education, not in training, and not in employment. | Numeric | Proportion (0–1) | Main analytical indicator | Variable abbreviation used in the manuscript: NEET_15_24. |
| Pobreza por ingresos | POB_ing | Income poverty rate | Percentage of the population living below the official income poverty line. | Numeric | Proportion (0–1) | Main analytical indicator | Variable abbreviation used in the manuscript: POB_ing. |
| Pobreza extrema por ingresos | POB_ext | Extreme income poverty rate | Percentage of the population living below the official extreme income poverty line. | Numeric | Proportion (0–1) | Main analytical indicator | Variable abbreviation used in the manuscript: POB_ext. |
| Pobreza por NBI | POB_NBI | Poverty by unsatisfied basic needs | Percentage of the population living in households with at least one unsatisfied basic need according to the official NBI approach. | Numeric | Proportion (0–1) | Contextual poverty indicator | Included in the dataset as an additional territorial poverty indicator. |
| Pobreza multidimensional | PM | Multidimensional poverty rate | Percentage of the population experiencing simultaneous deprivations according to the Alkire–Foster/INEC methodology. | Numeric | Proportion (0–1) | Main analytical indicator | Variable abbreviation used in the manuscript: PM. |
| Pobreza extrema multidimensional | PM_ext | Extreme multidimensional poverty rate | Percentage of the population with a deprivation intensity above the official extreme multidimensional poverty threshold. | Numeric | Proportion (0–1) | Main analytical indicator | Variable abbreviation used in the manuscript: PM_ext. |
| PEA | PEA | Economically active population | Number of people in the labour force, including employed and unemployed individuals. | Numeric | Count of persons | Population denominator / contextual variable | Used to describe the labour-force structure. |
| PEI | PEI | Economically inactive population | Number of working-age people who are not employed and are not actively seeking employment. | Numeric | Count of persons | Population denominator / contextual variable | Used to describe the labour-force structure. |
| PET | PET | Working-age population | Number of people classified as being of working age according to the official labour-statistics framework. | Numeric | Count of persons | Population denominator / contextual variable | Used as the denominator for labour-force participation indicators. |
| Media de ingreso laboral | ING | Average labour income | Average monthly labour income of employed individuals, expressed in current United States dollars. | Numeric | Current USD | Main analytical indicator | Variable abbreviation used in the manuscript: ING. |

# README content for repository

**README — Minimal dataset for PLOS ONE submission PONE-D-26-05632R1**

## Manuscript

Title: Mapping Territorial Labour-Related Well-Being Inequalities in Ecuador: A Multivariate Geometric Approach

Author: Bill Jonathan Serrano Orellana

Journal: PLOS ONE

Submission ID: PONE-D-26-05632R1

## Purpose of this repository

This repository contains the minimal dataset necessary to replicate the empirical results reported in the manuscript. The dataset was prepared from publicly available official labour and poverty statistics for Ecuador and organized for territorial multivariate analysis.

The analytical unit is the province-year. The dataset covers the 24 provinces of Ecuador for the period 2021-2024.

## Repository contents

This repository contains the following files:

- S1 Dataset.xlsx: analytical dataset used in the study.
- S2_Codebook.docx: human-readable variable dictionary.
- codebook_variables.csv: machine-readable variable dictionary.
- README.md: description of the dataset structure, variables, measurement units, and replication notes.

## Dataset structure

S1 Dataset.xlsx is an Excel workbook with four sheets:

- 2021
- 2022
- 2023
- 2024

Each sheet contains:

- 24 rows, corresponding to Ecuadorian provinces.
- 21 columns, corresponding to territorial labour-market, poverty, income, and population indicators.

The first column identifies the province. The remaining columns contain numeric indicators.

## Unit of analysis

Each observation represents one Ecuadorian province in one year. When the four annual sheets are combined, the complete analytical panel contains 96 province-year observations:

24 provinces x 4 years = 96 observations.

## Measurement scale

Most rate variables are expressed as proportions between 0 and 1. For example, a value of 0.413 represents 41.3%.

Population variables are expressed as counts of persons.

Average labour income is expressed in current United States dollars (USD).

## Main analytical variables used in the manuscript

The main multivariate analysis reported in the manuscript focuses on the following indicators:

| **Abbreviation** | **Dataset field** | **Full name** |
| --- | --- | --- |
| TPG | Tasa de participación global | Overall labour force participation rate |
| DES | Desempleo | Unemployment rate |
| EA | Empleo adecuado | Adequate employment rate |
| SUB | Subempleo | Underemployment rate |
| FOR | Sector formal | Formal employment share |
| NEET_15_24 | Personas que no estudian ni trabajan | NEET rate, ages 15-24 |
| POB_ing | Pobreza por ingresos | Income poverty rate |
| POB_ext | Pobreza extrema por ingresos | Extreme income poverty rate |
| PM | Pobreza multidimensional | Multidimensional poverty rate |
| PM_ext | Pobreza extrema multidimensional | Extreme multidimensional poverty rate |
| ING | Media de ingreso laboral | Average labour income |

Additional variables are retained in the dataset as contextual labour-market, poverty, and population indicators.

## Variable dictionary

A full variable dictionary is provided in S2_Codebook.docx and codebook_variables.csv. The dictionary includes the original dataset field name, the short abbreviation used in the manuscript, the English full name, the definition, the variable type, the unit or scale, and the analytical role.

## Data preparation notes

The dataset preserves the original annual structure used in the analysis. For replication, researchers may either:

1. work directly with each annual sheet, or

2. append the four sheets into a long-format province-year dataset by adding a year column.

For the multivariate analyses, rate indicators should be interpreted as proportions. If percentages are required for reporting, multiply the values by 100.

For methods that are sensitive to scale, such as PCA, HJ-Biplot, or Partial Triadic Analysis, variables should be centered and/or standardized according to the analytical specification described in the manuscript.

## Suggested replication workflow

1. Import S1 Dataset.xlsx.

2. Read the sheets 2021, 2022, 2023, and 2024.

3. Add a year column to each sheet.

4. Append the annual sheets into a single province-year dataset.

5. Select the 11 main analytical variables listed above.

6. Apply the preprocessing described in the manuscript.

7. Reproduce the descriptive tables, HJ-Biplot figures, and multivariate results.

## Missing data

Users should verify missing values after importing the workbook into their statistical software. The dataset was prepared as a cleaned analytical file for replication of the reported results.

## Source and provenance

The indicators correspond to official Ecuadorian labour, income, and poverty measures derived from public national statistical sources. The deposited file is the processed analytical dataset used in the manuscript, not the raw household-level microdata.

## Contact

For questions about the dataset or replication materials, please contact:

Bill Jonathan Serrano Orellana

Universidad Técnica de Machala

Email: bjserrano@utmachala.edu.ec
