# Supplementary material for: Mapping territorial labour-related well-being inequalities in Ecuador: A multivariate geometric approach
Source: PLoS One. 2026 Aug 31;21(8):e0343199. doi: 10.1371/journal.pone.0343199 (PMC13529017; doi:10.1371/journal.pone.0343199)
Supplement: Supplementary file 2 [file pone.0343199.s004.docx]

README — Minimal dataset for PLOS ONE submission PONE-D-26-05632R2

# Manuscript

**Title:** Mapping Territorial Labour-Related Well-Being Inequalities in Ecuador: A Multivariate Geometric Approach

**Author:** Bill Jonathan Serrano Orellana

**Journal:** PLOS ONE

**Submission ID:** PONE-D-26-05632R2

# Purpose of this repository

This repository contains the minimal dataset necessary to replicate the empirical results reported in the manuscript. The dataset was prepared from publicly available official labour and poverty statistics for Ecuador and organized for territorial multivariate analysis.

The analytical unit is the **province-year**. The dataset covers the 24 provinces of Ecuador for the period **2021–2024**.

# Repository contents

This repository contains the following files:

- S1 Dataset.xlsx: analytical dataset used in the study.
- S2 Codebook.docx: human-readable variable dictionary.
- codebook_variables.csv: machine-readable variable dictionary.
- S4 README.docx: description of the dataset structure, variables, measurement units, and replication notes.

# Dataset structure

S1 Dataset.xlsx is an Excel workbook with four sheets:

- 2021
- 2022
- 2023
- 2024

Each sheet contains:

- 24 rows, corresponding to Ecuadorian provinces.
- 21 columns, corresponding to territorial labour-market, poverty, income, and population indicators.

The first column identifies the province. The remaining columns contain numeric indicators.

# Unit of analysis

Each observation represents one Ecuadorian province in one year. When the four annual sheets are combined, the complete analytical panel contains 96 province-year observations:

24 provinces × 4 years = 96 observations.

# Measurement scale

Most rate variables are expressed as proportions between 0 and 1. For example, a value of 0.413 represents 41.3%.

Population variables are expressed as counts of persons.

Average labour income is expressed in current United States dollars (USD).

# Main analytical variables used in the manuscript

The main multivariate analysis reported in the manuscript focuses on the following indicators:

| **Abbreviation** | **Dataset field** | **Full name** |
| --- | --- | --- |
| TPG | Tasa de participación global | Overall labour force participation rate |
| DES | Desempleo | Unemployment rate |
| EA | Empleo adecuado | Adequate employment rate |
| SUB | Subempleo | Underemployment rate |
| FOR | Sector formal | Formal employment share |
| NEET_15_24 | Personas que no estudian ni trabajan | NEET rate, ages 15–24 |
| POB_ing | Pobreza por ingresos | Income poverty rate |
| POB_ext | Pobreza extrema por ingresos | Extreme income poverty rate |
| PM | Pobreza multidimensional | Multidimensional poverty rate |
| PM_ext | Pobreza extrema multidimensional | Extreme multidimensional poverty rate |
| ING | Media de ingreso laboral | Average labour income |

Additional variables are retained in the dataset as contextual labour-market, poverty, and population indicators.

# Variable dictionary

A full variable dictionary is provided in S2 Codebook.docx and codebook_variables.csv. The dictionary includes the original dataset field name, the short abbreviation used in the manuscript, the English full name, the definition, the variable type, the unit or scale, and the analytical role.

# Data preparation notes

The dataset preserves the original annual structure used in the analysis. For replication, researchers may either:

1. work directly with each annual sheet, or

2. append the four sheets into a long-format province-year dataset by adding a year column.

For the multivariate analyses, rate indicators should be interpreted as proportions. If percentages are required for reporting, multiply the values by 100.

For methods that are sensitive to scale, such as PCA, HJ-Biplot, or Partial Triadic Analysis, variables should be centered and/or standardized according to the analytical specification described in the manuscript.

# Suggested replication workflow

1. Import S1 Dataset.xlsx.

2. Read the sheets 2021, 2022, 2023, and 2024.

3. Add a year column to each sheet.

4. Append the annual sheets into a single province-year dataset.

5. Select the 11 main analytical variables listed above.

6. Apply the preprocessing described in the manuscript.

7. Reproduce the descriptive tables, HJ-Biplot figures, and multivariate results.

# Missing data

Users should verify missing values after importing the workbook into their statistical software. The dataset was prepared as a cleaned analytical file for replication of the reported results.

# Source and provenance

The indicators correspond to official Ecuadorian labour, income, and poverty measures derived from public national statistical sources. The deposited file is the processed analytical dataset used in the manuscript, not the raw household-level microdata.

# Contact

For questions about the dataset or replication materials, please contact:

Bill Jonathan Serrano Orellana

Universidad Técnica de Machala

Email: bjserrano@utmachala.edu.ec
