# Supplementary material for: Mapping territorial labour-related well-being inequalities in Ecuador: A multivariate geometric approach
Source: PLoS One. 2026 Aug 31;21(8):e0343199. doi: 10.1371/journal.pone.0343199 (PMC13529017; doi:10.1371/journal.pone.0343199)
Supplement: Supplementary file 3 [file pone.0343199.s006.docx]

**Response to Reviewers**

**Manuscript ID:** PONE-D-26-05632R1

**Title:** *Mapping Territorial Labour-Related Well-Being Inequalities in Ecuador: A Multivariate Geometric Approach*

Dear Academic Editor and Reviewers,

I sincerely thank you for your careful reading of my manuscript and for the constructive and insightful comments. I have revised the manuscript thoroughly and believe that the new version has been substantially improved in structure, clarity, and reproducibility. Below, I provide a point-by-point response to the editor’s requirements and to each reviewer’s comments.

**Response to the Academic Editor**

I am grateful for the editor’s guidance and have implemented the requested revisions.

**1. Please ensure that the manuscript complies with PLOS ONE style requirements.**

**Response:** The manuscript has been revised to improve consistency with PLOS ONE style requirements. I reviewed headings, figure and table references, section structure, English-language wording, and the presentation of the final manuscript. In addition, I ensured that the revised submission files follow the journal’s naming and formatting requirements.

**2. Please ensure that Figures 1, 10, 11, 12, and 13 are referred to in the text.**

**Response:** I have now explicitly cited Figures 1, 10, 11, 12, and 13 in the main text at the appropriate points in the Methods, Results, and Discussion sections.

**3. Please include a copy of Table 1 referred to in the text.**

**Response:** Table 1 has been included in the revised manuscript and is explicitly referenced in the Materials and Methods section.

**4. Please ensure that Tables 3, 4, and 9 are referred to in the text.**

**Response:** I have incorporated explicit in-text references to Tables 3, 4, and 9 in the relevant Results and Discussion subsections.

**Reference list check.**

**Response:** I reviewed the reference list and corrected it for completeness and consistency with the citations used in the revised manuscript. Any revisions to the references were made in accordance with the editor’s request.

**Additional technical correction requested by the Editorial Office**

In response to the Editorial Office’s technical request, I have removed the previous manuscript file and reattached the revised manuscript in an accepted editable file format (.docx). I have also ensured that the Data Availability Statement in the manuscript includes the Zenodo DOI for the public repository containing the minimal dataset.

**Response to Reviewer 1**

I thank Reviewer 1 for the positive assessment of the manuscript and for the helpful recommendation.

**Comment:** The manuscript is well developed, but some titles are in a language other than English and may not be understandable to all readers.
**Response:** I appreciate this observation. I carefully reviewed the manuscript and corrected residual non-English headings, titles, and captions in order to ensure consistency and accessibility for an international readership.

**Response to Reviewer 2**

I am very grateful for this detailed and constructive review. The comments were extremely helpful in improving the manuscript. I addressed each point as follows.

**1. The manuscript should clearly separate Materials and Methods, Results, and Discussion, and include an independent conclusion.**

**Response:** I revised the structure of the manuscript accordingly. Section 3 is devoted to Materials and Methods, Section 4 now presents the empirical results in a descriptive way, Section 5 develops the discussion and policy implications, and an independent Section 6 has been added to provide a concise conclusion.

**2. Sections 3.2–3.3 are too extensive and complex; reduce redundancy and emphasize methodological suitability rather than textbook-level detail.**

**Response:** I revised Sections 3.2–3.3 to improve clarity and reduce redundancy. While I retained the core mathematical elements necessary for transparency and interpretability, I streamlined the explanatory text and placed greater emphasis on why HJ-biplot and PTA are appropriate for the present study, namely the three-way structure of the data, the joint representation of provinces and indicators, and the synthesis of temporal structure through the compromise space.

**3. Avoid residual Spanish and write all titles and phrases in English.**

**Response:** I carefully reviewed the manuscript and corrected the remaining Spanish-language elements in section titles, figure captions, and the main text.

**4. Briefly acknowledge that 2020 was excluded because it corresponds to the pandemic shock and comparability problems.**

**Response:** I incorporated an explicit justification in the Materials and Methods section. The revised manuscript now explains that 2020 was excluded because it reflects the acute pandemic shock and exceptionally disrupted labour-market conditions, which may reduce interannual comparability. The study therefore focuses on the 2021–2024 post-pandemic recovery phase.

**5. Justify the use of HJ-Biplot and PTA instead of panel econometrics or spatial autocorrelation methods.**

**Response:** I added a specific methodological justification in the Materials and Methods section. The revised text now clarifies that conventional panel or spatial econometric methods are well suited to testing specific causal hypotheses, whereas the purpose of this study is exploratory and structural: to summarize the joint geometry of eleven interrelated indicators across 24 provinces and four annual slices, and to compare annual territorial trajectories within a common factorial space. I therefore present the HJ-biplot–PTA framework as complementary to, rather than a substitute for, conventional econometric analysis.

**6. Include a subsection on limitations, acknowledging the small provincial sample and the fact that provincial averages may hide intraprovincial disparities.**

**Response:** I incorporated a dedicated Limitations subsection in the Discussion. This section now explicitly acknowledges that the analysis is based on 24 provincial units and that provincial averages may conceal important within-province disparities, including urban–rural differences and internal heterogeneity in provinces such as Pichincha and Guayas.

**Data availability**

In response to the journal’s data availability requirements, I have updated the Data Availability Statement and deposited the minimal dataset necessary to replicate the study findings in Zenodo. The dataset is publicly available at: https://doi.org/10.5281/zenodo.19839634.

The repository record includes the processed province-year analytical dataset for Ecuadorian provinces during 2021–2024, the variable dictionary, and supporting documentation necessary to reproduce the results reported in the manuscript. The revised manuscript now states that the deposited dataset corresponds to the processed analytical data used in this study, while the original official ENEMDU microdata, statistical tabulations, and methodological documents are publicly available from Ecuador’s National Institute of Statistics and Censuses (INEC).

**Final statement**

I sincerely thank the editor and both reviewers for their valuable comments. I believe that the revised manuscript has been significantly strengthened as a result of this process, and I hope that the new version is now suitable for publication in *PLOS ONE*.

Sincerely,
**Bill Serrano-Orellana**
