# Supplementary material for: Mapping territorial labour-related well-being inequalities in Ecuador: A multivariate geometric approach
Source: PLoS One. 2026 Aug 31;21(8):e0343199. doi: 10.1371/journal.pone.0343199 (PMC13529017; doi:10.1371/journal.pone.0343199)
Supplement: Supplementary file 4 [file pone.0343199.s007.docx]

**Response to Reviewers**

**Manuscript ID:** PONE-D-26-05632R1

**Title:** Mapping Territorial Labour-Related Well-Being Inequalities in Ecuador: A Multivariate Geometric Approach

**Journal:** PLOS ONE

Dear Academic Editor and Reviewers,

I sincerely thank the Academic Editor and the reviewers for their careful assessment of my revised manuscript. I am grateful that both reviewers considered that the previous comments had been addressed and that the manuscript is technically sound, the data support the conclusions, and the statistical analysis has been performed appropriately.

In response to the remaining concern, I have carefully revised the entire manuscript for English language quality, grammar, syntax, punctuation, word choice, readability, and consistency of terminology. I also checked the manuscript for consistent use of key terms such as labour-related well-being, labour market, underemployment, formality, NEET, multidimensional poverty, HJ-biplot, and Partial Triadic Analysis. These revisions were made to improve the accuracy and clarity of expression without altering the data, methodology, results, or substantive conclusions of the article.

A marked-up version of the manuscript showing the changes and a clean version of the revised manuscript have been uploaded as requested.

**Point-by-point response**

**1. Academic Editor**

Comment: “Please make a language check of the paper. As also recommended by the reviewer, I consider that the paper should be read by a native English speaker to ensure the accuracy of expression.”

Response: I thank the Academic Editor for this recommendation. The entire manuscript has been carefully reviewed and edited for English language quality, clarity, grammar, syntax, punctuation, and consistency of academic expression. Particular attention was given to improving sentence structure, reducing ambiguity, and harmonising terminology throughout the manuscript.

Changes made: The full manuscript was revised for language and clarity. The revisions are visible in the tracked-changes version and incorporated in the clean version. No substantive changes were made to the study design, data, statistical analysis, results, or conclusions.

**2. Reviewer #1**

Comment: “All comments have been addressed.”

Response: I sincerely thank Reviewer #1 for the positive assessment of the revised manuscript.

Changes made: No further substantive changes were required in response to this comment.

**3. Reviewer #2**

Comment: “All comments have been addressed.”

Response: I sincerely thank Reviewer #2 for confirming that the previous comments have been addressed.

Changes made: No further substantive changes were required in response to this comment.

**4. Reviewer #2**

Comment: “Language check of entire manuscript is required.”

Response: I thank Reviewer #2 for this helpful recommendation. In response, the entire manuscript has been reviewed for English language accuracy and readability. I corrected grammatical and typographical issues, improved sentence flow, clarified expressions where necessary, and checked the consistency of terminology across the Abstract, Introduction, Literature Review, Materials and Methods, Results, Discussion, Conclusion, Data Availability Statement, Supporting Information, and References.

Changes made: Language and presentation edits were made throughout the manuscript. These changes are shown in the revised manuscript with tracked changes. The clean manuscript includes the same language revisions without markup.

I hope that the revised manuscript now meets the language and presentation requirements of PLOS ONE.

Kind regards,

Bill Serrano-Orellana, PhD
