# Supplementary material for: Mapping territorial labour-related well-being inequalities in Ecuador: A multivariate geometric approach
Source: PLoS One. 2026 Aug 31;21(8):e0343199. doi: 10.1371/journal.pone.0343199 (PMC13529017; doi:10.1371/journal.pone.0343199)
Supplement: Peer review history

# Peer review: Decision Letter 0

**DOI:** 10.1371/journal.pone.0343199.r001

**Authors:** Valentina Diana Rusu

14 Apr 2026

Dear Dr. Serrano Orellana,

Thank you for submitting your manuscript to PLOS ONE. After careful consideration, we feel that it has merit but does not fully meet PLOS ONE’s publication criteria as it currently stands. Therefore, we invite you to submit a revised version of the manuscript that addresses the points raised during the review process.

==============================

**ACADEMIC EDITOR:** Please take the reviewers' recommendations into account and make changes to the paper accordingly.

==============================

Please submit your revised manuscript by May 29 2026 11:59PM. If you will need more time than this to complete your revisions, please reply to this message or contact the journal office at plosone@plos.org. When you're ready to submit your revision, log on to https://www.editorialmanager.com/pone/ and select the 'Submissions Needing Revision' folder to locate your manuscript file.

- A letter that responds to each point raised by the academic editor and reviewer(s). You should upload this letter as a separate file labeled 'Response to Reviewers'.
- A marked-up copy of your manuscript that highlights changes made to the original version. You should upload this as a separate file labeled 'Revised Manuscript with Track Changes'.
- An unmarked version of your revised paper without tracked changes. You should upload this as a separate file labeled 'Manuscript'.

If you would like to make changes to your financial disclosure, please include your updated statement in your cover letter. Guidelines for resubmitting your figure files are available below the reviewer comments at the end of this letter.

If applicable, we recommend that you deposit your laboratory protocols in protocols.io to enhance the reproducibility of your results. Protocols.io assigns your protocol its own identifier (DOI) so that it can be cited independently in the future. For instructions see: https://journals.plos.org/plosone/s/submission-guidelines#loc-laboratory-protocols. Additionally, PLOS ONE offers an option for publishing peer-reviewed Lab Protocol articles, which describe protocols hosted on protocols.io. Read more information on sharing protocols at https://plos.org/protocols?utm_medium=editorial-email&utm_source=authorletters&utm_campaign=protocols.

As the corresponding author, your ORCID iD is verified in the submission system and will appear in the published article. PLOS supports the use of ORCID, and we encourage all coauthors to register for an ORCID iD and use it as well. Please encourage your coauthors to verify their ORCID iD within the submission system before final acceptance, as unverified ORCID iDs will not appear in the published article. *Only*  the individual author can complete the verification step; PLOS staff *cannot*  verify ORCID iDs on behalf of authors.

We look forward to receiving your revised manuscript.

Kind regards,

Valentina Diana Rusu, PhD

Academic Editor

PLOS One

Journal Requirements:

When submitting your revision, we need you to address these additional requirements.

1. Please ensure that your manuscript meets PLOS ONE's style requirements, including those for file naming. The PLOS ONE style templates can be found at

https://journals.plos.org/plosone/s/file?id=wjVg/PLOSOne_formatting_sample_main_body.pdf and https://journals.plos.org/plosone/s/file?id=ba62/PLOSOne_formatting_sample_title_authors_affiliations.pdf

2. Please ensure that you refer to Figures 1, 10, 11, 12, and 13 in your text as, if accepted, production will need this reference to link the reader to the figures.

3. Please include a copy of Table 1 to which you refer to in your text on pages 14 and 34.

4. We note you have included a table to which you do not refer in the text of your manuscript. Please ensure that you refer to Tables 3, 4, and 9 in your text; if accepted, production will need this reference to link the reader to the tables.

If the reviewer comments include a recommendation to cite specific previously published works, please review and evaluate these publications to determine whether they are relevant and should be cited. There is no requirement to cite these works unless the editor has indicated otherwise.

Please review your reference list to ensure that it is complete and correct. If you have cited papers that have been retracted, please include the rationale for doing so in the manuscript text, or remove these references and replace them with relevant current references. Any changes to the reference list should be mentioned in the rebuttal letter that accompanies your revised manuscript. If you need to cite a retracted article, indicate the article’s retracted status in the References list and also include a citation and full reference for the retraction notice.

[Note: HTML markup is below. Please do not edit.]

Reviewers' comments:

Reviewer's Responses to Questions

**Comments to the Author**

1. Is the manuscript technically sound, and do the data support the conclusions?

Reviewer #1: Yes

Reviewer #2: Yes

**********

2. Has the statistical analysis been performed appropriately and rigorously?

Reviewer #1: Yes

Reviewer #2: Yes

**********

3. Have the authors made all data underlying the findings in their manuscript fully available?

The PLOS Data policy

Reviewer #1: No

Reviewer #2: Yes

**********

4. Is the manuscript presented in an intelligible fashion and written in standard English?

Reviewer #1: Yes

Reviewer #2: Yes

**********

Reviewer #1: The paper addresses an intriguing topic and manages it proficiently. The arguments are coherently organized, and the methodology employed appears both relevant and suitable. The analysis of results, discussion, relevance to existing literature, and acknowledgment of limitations are also effectively executed. However, I have a minor suggestion: some headings are in a language other than English and therefore may not be understandable to all readers.

Reviewer #2: The manuscript is methodologically and topic-wise a good work. It employs a sound econometric model and techniques. It is good that it uses official data from Ecuador's National Institute of Statistics and Censuses for the years 2021–2024. The emphasis is on spatial disparities in employment-related well-being, encompassing job quantity and quality, income, poverty (both monetary and multidimensional), and youth not in education, employment, or training. There seem to be few, if any, published studies that have utilized this specific HJ-Biplot and PTA combination to analyze post-pandemic labor wellness at the provincial level in Ecuador.

(1) According to my knowledge, PLOS ONE requires a structured manuscript comprising separate sections for Materials and Methods, Results, and Discussion (or a combined Results & Discussion). The current version includes "3. Materials and Methods" (acceptable), but "4. Resultados" (Use English vocabulary please) conflates results with some interpretation, while "5. Discussion" persists in interpretation and policy. Clearly delineate these sections. Relocate purely descriptive findings (tables, figures, axis loadings, province positions, trajectories) to the Results section. Maintain substantial interpretation, literary connections, and policy within the Discussion section. Include a concise, independent conclusion that encapsulates the primary insights.

(2) Sections 3.2–3.3 are extensive and complex, containing complete SVD/HJ formulas and reiterated definitions. Although transparency is beneficial, readers of PLOS ONE, who represent a broad interdisciplinary audience, may perceive it as excessive. Eliminate redundancy (e.g., transfer whole formulas to an auxiliary document if necessary). Highlight the appropriateness of these methodologies for the data and inquiries (three-way structure, visual joint representation, temporal synthesis) rather than focusing on textbook-level specifics.

(3) Avoid Spanish remnants. Write all phrases/titles etc in English.

(4) Period 2021–2024 is post-pandemic recovery; briefly acknowledge 2020 shock was excluded due to data comparability or extreme values.

(5) The author should specifically justify the selection of HJ-Biplot and PTA over conventional panel data econometrics or Spatial Autocorrelation methods.

(6) The research employs provincial-level data (N=24). Although statistically valid for Biplots, the sample size is inadequate for specific forms of multivariate stability. Incorporate a "Limitations" subsection. Recognize that provincial averages may obscure intra-provincial (urban versus rural) disparities, which are prominent in provinces such as Guayas or Pichincha. For instance, in Pichincha, the urban core may have elevated "Adequate Employment" rates, yet the surrounding rural sections may experience severe "Multidimensional Poverty."

**********

what does this mean?). If published, this will include your full peer review and any attached files.

If you choose “no”, your identity will remain anonymous but your review may still be made public.

**Do you want your identity to be public for this peer review?**  For information about this choice, including consent withdrawal, please see our Privacy Policy

Reviewer #1: No

Reviewer #2: **Yes:** AYESHA NAZUK

**********

[NOTE: If reviewer comments were submitted as an attachment file, they will be attached to this email and accessible via the submission site. Please log into your account, locate the manuscript record, and check for the action link "View Attachments". If this link does not appear, there are no attachment files.]

To ensure your figures meet our technical requirements, please review our figure guidelines: https://journals.plos.org/plosone/s/figures

You may also use PLOS’s free figure tool, NAAS, to help you prepare publication quality figures: https://journals.plos.org/plosone/s/figures#loc-tools-for-figure-preparation.

NAAS will assess whether your figures meet our technical requirements by comparing each figure against our figure specifications.

# Author comment: Author response to Decision Letter 1

**DOI:** 10.1371/journal.pone.0343199.r002

28 Apr 2026

Please see the attached “Response to Reviewers” document for the full point-by-point response to the Academic Editor’s and reviewers’ comments.

In summary, I revised the manuscript to improve compliance with PLOS ONE style requirements; added explicit in-text references to Figures 1, 10, 11, 12, and 13 and to Tables 1, 3, 4, and 9; reviewed and corrected the reference list; and ensured consistency in English throughout the manuscript.

I also revised the manuscript structure to clearly separate Materials and Methods, Results, Discussion, and an independent Conclusion. In response to Reviewer 2, I clarified the exclusion of 2020, justified the use of HJ-biplot and PTA relative to conventional panel and spatial econometric approaches, streamlined Sections 3.2–3.3, and added a dedicated Limitations subsection.

In addition, I addressed the Editorial Office’s technical requests. I reuploaded the manuscript in an accepted editable file format (.docx), updated the Data Availability Statement, and deposited the minimal dataset necessary to replicate the study findings in Zenodo. The dataset is publicly available at: https://doi.org/10.5281/zenodo.19839634. The repository record includes the processed province-year analytical dataset, the variable dictionary, and supporting documentation necessary to reproduce the results reported in the manuscript.

Attachment Submitted filename: Response to Reviewers.docx

# Peer review: Decision Letter 1

**DOI:** 10.1371/journal.pone.0343199.r003

**Authors:** Valentina Diana Rusu

14 May 2026

Dear Dr. Serrano Orellana,

Thank you for submitting your manuscript to PLOS ONE. After careful consideration, we feel that it has merit but does not fully meet PLOS ONE’s publication criteria as it currently stands. Therefore, we invite you to submit a revised version of the manuscript that addresses the points raised during the review process.

==============================

**ACADEMIC EDITOR:**

==============================

Please submit your revised manuscript by Jun 28 2026 11:59PM. If you will need more time than this to complete your revisions, please reply to this message or contact the journal office at plosone@plos.org. When you're ready to submit your revision, log on to https://www.editorialmanager.com/pone/ and select the 'Submissions Needing Revision' folder to locate your manuscript file.

- A letter that responds to each point raised by the academic editor and reviewer(s). You should upload this letter as a separate file labeled 'Response to Reviewers'.
- A marked-up copy of your manuscript that highlights changes made to the original version. You should upload this as a separate file labeled 'Revised Manuscript with Track Changes'.
- An unmarked version of your revised paper without tracked changes. You should upload this as a separate file labeled 'Manuscript'.

If you would like to make changes to your financial disclosure, please include your updated statement in your cover letter. Guidelines for resubmitting your figure files are available below the reviewer comments at the end of this letter.

If applicable, we recommend that you deposit your laboratory protocols in protocols.io to enhance the reproducibility of your results. Protocols.io assigns your protocol its own identifier (DOI) so that it can be cited independently in the future. For instructions see: https://journals.plos.org/plosone/s/submission-guidelines#loc-laboratory-protocols. Additionally, PLOS ONE offers an option for publishing peer-reviewed Lab Protocol articles, which describe protocols hosted on protocols.io. Read more information on sharing protocols at https://plos.org/protocols?utm_medium=editorial-email&utm_source=authorletters&utm_campaign=protocols.

As the corresponding author, your ORCID iD is verified in the submission system and will appear in the published article. PLOS supports the use of ORCID, and we encourage all coauthors to register for an ORCID iD and use it as well. Please encourage your coauthors to verify their ORCID iD within the submission system before final acceptance, as unverified ORCID iDs will not appear in the published article. *Only*  the individual author can complete the verification step; PLOS staff *cannot*  verify ORCID iDs on behalf of authors.

We look forward to receiving your revised manuscript.

Kind regards,

Valentina Diana Rusu, PhD

Academic Editor

PLOS One

Journal Requirements:

If the reviewer comments include a recommendation to cite specific previously published works, please review and evaluate these publications to determine whether they are relevant and should be cited. There is no requirement to cite these works unless the editor has indicated otherwise.

Please review your reference list to ensure that it is complete and correct. If you have cited papers that have been retracted, please include the rationale for doing so in the manuscript text, or remove these references and replace them with relevant current references. Any changes to the reference list should be mentioned in the rebuttal letter that accompanies your revised manuscript. If you need to cite a retracted article, indicate the article’s retracted status in the References list and also include a citation and full reference for the retraction notice.

[Note: HTML markup is below. Please do not edit.]

Reviewers' comments:

Reviewer's Responses to Questions

**Comments to the Author**

Reviewer #1: All comments have been addressed

Reviewer #2: All comments have been addressed

**********

2. Is the manuscript technically sound, and do the data support the conclusions?

Reviewer #1: Yes

Reviewer #2: Yes

**********

3. Has the statistical analysis been performed appropriately and rigorously?

Reviewer #1: (No Response)

Reviewer #2: Yes

**********

4. Have the authors made all data underlying the findings in their manuscript fully available?

The PLOS Data policy

Reviewer #1: Yes

Reviewer #2: Yes

**********

5. Is the manuscript presented in an intelligible fashion and written in standard English?

Reviewer #1: Yes

Reviewer #2: No

**********

Reviewer #1: (No Response)

Reviewer #2: Language check of entire manuscript is required.

Language check of entire manuscript is required.

**********

what does this mean?). If published, this will include your full peer review and any attached files.

If you choose “no”, your identity will remain anonymous but your review may still be made public.

**Do you want your identity to be public for this peer review?**  For information about this choice, including consent withdrawal, please see our Privacy Policy

Reviewer #1: No

Reviewer #2: **Yes:** AYESHA NAZUK

**********

[NOTE: If reviewer comments were submitted as an attachment file, they will be attached to this email and accessible via the submission site. Please log into your account, locate the manuscript record, and check for the action link "View Attachments". If this link does not appear, there are no attachment files.]

To ensure your figures meet our technical requirements, please review our figure guidelines: https://journals.plos.org/plosone/s/figures

You may also use PLOS’s free figure tool, NAAS, to help you prepare publication quality figures: https://journals.plos.org/plosone/s/figures#loc-tools-for-figure-preparation.

NAAS will assess whether your figures meet our technical requirements by comparing each figure against our figure specifications.

# Author comment: Author response to Decision Letter 2

**DOI:** 10.1371/journal.pone.0343199.r004

25 May 2026

Dear Academic Editor and Reviewers,

I sincerely thank the Academic Editor and the reviewers for their careful assessment of my revised manuscript. I am grateful that both reviewers considered that the previous comments had been addressed and that the manuscript is technically sound, the data support the conclusions, and the statistical analysis has been performed appropriately.

In response to the remaining concern, the entire manuscript has undergone a full English-language review focused on accuracy of expression, clarity, grammar, syntax, punctuation, word choice, readability, and consistency of academic terminology. These revisions were made to improve the accuracy and clarity of expression without altering the data, methodology, results, or substantive conclusions of the article.

Point-by-point response

1. Academic Editor

Comment: “Please make a language check of the paper. As also recommended by the reviewer, I consider that the paper should be read by a native English speaker to ensure the accuracy of expression.”

Response: I thank the Academic Editor for this recommendation. The entire manuscript has undergone a full English-language review focused on accuracy of expression, clarity, grammar, syntax, punctuation, word choice, readability, and consistency of academic terminology. Particular attention was given to improving sentence structure, reducing ambiguity, and harmonising terminology throughout the manuscript.

Changes made: The full manuscript was revised for language and clarity. The revisions are visible in the tracked-changes version and incorporated in the clean version. No substantive changes were made to the study design, data, statistical analysis, results, or conclusions.

2. Reviewer #1

Comment: “All comments have been addressed.”

Response: I sincerely thank Reviewer #1 for the positive assessment of the revised manuscript.

Changes made: No further substantive changes were required in response to this comment.

3. Reviewer #2

Comment: “All comments have been addressed.”

Response: I sincerely thank Reviewer #2 for confirming that the previous comments have been addressed.

Changes made: No further substantive changes were required in response to this comment.

4. Reviewer #2

Comment: “Language check of entire manuscript is required.”

Response: I thank Reviewer #2 for this helpful recommendation. In response, the entire manuscript has undergone a full English-language review for accuracy of expression and readability. I corrected grammatical and typographical issues, improved sentence flow, clarified expressions where necessary, and checked the consistency of terminology across the Abstract, Introduction, Literature Review, Materials and Methods, Results, Discussion, Conclusion, Data Availability Statement, Supporting Information, and References.

Changes made: Language and presentation edits were made throughout the manuscript. These changes are shown in the revised manuscript with tracked changes. The clean manuscript includes the same language revisions without markup.

I hope that the revised manuscript now meets the language and presentation requirements of PLOS ONE.

Kind regards,

Bill Serrano-Orellana, PhD

Attachment Submitted filename: Response to Reviewers PLOS ONE.docx

# Editor report: Decision Letter 2

**DOI:** 10.1371/journal.pone.0343199.r005

**Authors:** Valentina Diana Rusu

12 Aug 2026

Mapping Territorial Labour-Related Well-Being Inequalities in Ecuador: A Multivariate Geometric Approach

PONE-D-26-05632R2

Dear Dr. Serrano Orellana,

We’re pleased to inform you that your manuscript has been judged scientifically suitable for publication and will be formally accepted for publication once it meets all outstanding technical requirements.

Within one week, you’ll receive an e-mail detailing the required amendments. When these have been addressed, you’ll receive a formal acceptance letter and your manuscript will be scheduled for publication.

An invoice will be generated when your article is formally accepted. Please note, if your institution has a publishing partnership with PLOS and your article meets the relevant criteria, all or part of your publication costs will be covered. Please make sure your user information is up-to-date by logging into Editorial Manager at Editorial Manager® and clicking the ‘Update My Information' link at the top of the page. For questions related to billing, please contact billing support.

If your institution or institutions have a press office, please notify them about your upcoming paper to help maximize its impact. If they’ll be preparing press materials, please inform our press team as soon as possible -- no later than 48 hours after receiving the formal acceptance. Your manuscript will remain under strict press embargo until 2 pm Eastern Time on the date of publication. For more information, please contact onepress@plos.org.

Kind regards,

Valentina Diana Rusu, PhD

Academic Editor

PLOS One

Additional Editor Comments (optional):

Reviewers' comments:

# Editor report: Acceptance letter

**DOI:** 10.1371/journal.pone.0343199.r006

**Authors:** Valentina Diana Rusu

PONE-D-26-05632R2

PLOS One

Dear Dr. Serrano Orellana,

I'm pleased to inform you that your manuscript has been deemed suitable for publication in PLOS One. Congratulations! Your manuscript is now being handed over to our production team.

At this stage, our production department will prepare your paper for publication. This includes ensuring the following:

* All references, tables, and figures are properly cited

* All relevant supporting information is included in the manuscript submission,

* There are no issues that prevent the paper from being properly typeset

You will receive further instructions from the production team, including instructions on how to review your proof when it is ready. Please keep in mind that we are working through a large volume of accepted articles, so please give us a few days to review your paper and let you know the next and final steps.

Lastly, if your institution or institutions have a press office, please let them know about your upcoming paper now to help maximize its impact. If they'll be preparing press materials, please inform our press team within the next 48 hours. Your manuscript will remain under strict press embargo until 2 pm Eastern Time on the date of publication. For more information, please contact onepress@plos.org.

You will receive an invoice from PLOS for your publication fee after your manuscript has reached the completed accept phase. If you receive an email requesting payment before acceptance or for any other service, this may be a phishing scheme. Learn how to identify phishing emails and protect your accounts at https://explore.plos.org/phishing.

If we can help with anything else, please email us at customercare@plos.org.

Thank you for submitting your work to PLOS One and supporting open access.

Kind regards,

PLOS ONE Editorial Office Staff

on behalf of

Dr. Valentina Diana Rusu

Academic Editor

PLOS One
